# MicroRNA Profiling in Wilms Tumor: Identification of Potential Biomarkers

**DOI:** 10.3389/fped.2020.00337

**Published:** 2020-07-16

**Authors:** Fabiola Jimena Pérez-Linares, Mario Pérezpeña-Diazconti, Jorge García-Quintana, Guillermina Baay-Guzmán, Lourdes Cabrera-Muñoz, Stanislaw Sadowinski-Pine, Carlos Serrano-Bello, Marco Murillo-Maldonado, Alejandra Contreras-Ramos, Pilar Eguía-Aguilar

**Affiliations:** ^1^Laboratorio de Biología Molecular, Departamento de Patología Clínica y Experimental, Hospital Infantil de México Federico Gómez, Ciudad de México, Mexico; ^2^Facultad de Química, Universidad Nacional Autónoma de México, Ciudad de México, Mexico; ^3^Instituto de Oftalmología Conde de Valenciana, Unidad de Investigación, Ciudad de México, Mexico; ^4^Unidad de Investigación en Enfermedades Oncológicas, Hospital Infantil de México Federico Gómez, Ciudad de México, México; ^5^Servicio de Onco-Hematología Pediátrica, Hospital Infantil de México Federico Gómez, Ciudad de México, Mexico; ^6^Laboratorio de Biología del Desarrollo y Teratogénesis Experimental, Hospital Infantil de México Federico Gómez, Ciudad de México, Mexico

**Keywords:** anaplasia, microRNA, triphasic, Wilms tumor, TLDA, *in situ* hybridization, RT-qPCR

## Abstract

Wilms tumor (WT) is the most frequently diagnosed malignant renal tumor in children. With current treatments, ~90% of children diagnosed with WT survive and generally present with tumors characterized by favorable histology (FHWT), whereas prognosis is poor for the remaining 10% of cases where the tumors are characterized by cellular diffuse anaplasia (DAWT). Relatively few studies have investigated microRNA-related epigenetic regulation and its relationship with altered gene expression in WT. Here, we aim to identify microRNAs differentially expressed in WT and describe their expression in terms of cellular anaplasia, metastasis, and association with the main genetic alterations in WT to identify potential prognostic biomarkers. Expression profiling using TaqMan low-density array was performed in a discovery cohort consisting of four DAWT and eight FHWT samples. Relative quantification resulted in the identification of 109 (48.7%) microRNAs differentially expressed in both WT types. Of these, miR-10a-5p, miR-29a-3p, miR-181a-5p, miR-200b-3p, and miR-218-5p were selected and tested by RT-qPCR on a validation cohort of 53 patient samples. MiR-29a and miR-218 showed significant differences in FHWT with low (*P* = 0.0018) and high (*P* = 0.0131) expression, respectively. To discriminate between miRNA expression FHWTs and healthy controls, the receiver operating characteristic (ROC) curves were obtained; miR-29a AUC was 0.7843. Furthermore, low expression levels of miR-29a and miR-200b (*P* = 0.0027 and *P* = 0.0248) were observed in metastatic tumors. ROC curves for miR-29a discriminated metastatic patients (AUC = 0.8529) and miR-200b (AUC = 0.7757). To confirm the differences between cases with poor prognosis, we performed *in situ* hybridization for three microRNAs in five DAWT and 17 FHWT samples, and only significant differences between adjacent tissues and FHWT tumors were found for miR-181a, miR-200b, and miR-218, in both total pixels and nuclear analyses. Analysis of copy number variation in genes showed that the most prevalent alterations were *WTX* (47%), *IGF2* (21%), 1q (36%) gain, 1p36 (16%), and *WTX* deletion/1q duplicate (26%). The five microRNAs evaluated are involved in the Hippo signaling pathway and participate in Wilms tumor development through their effects on differentiation, proliferation, angiogenesis, and metastasis.

## Introduction

Wilms tumor (WT), also known as nephroblastoma, is a malignant, solid kidney tumor that affects children. It accounts for ~8% of all infant neoplasia, and diagnosis usually occurs before the age of five (1). Approximately 90% of WT patients survive and are associated with favorable histology (FHWT); however, this percentage diminished to 75% in metastatic cases. On the other hand, the remaining 10% of WT present unfavorable histology; they are characterized by cellular diffuse anaplasia (DAWT) and poor prognosis (2, 3). Histologically, WT presents three types of cells: blastemal, mesenchymal, and epithelial, with the blastemal type being the most frequent, followed by triphasic, which is characterized by the presence of the three cell types in the same tumor (4). Each of these cell types has specific characteristics related to WT development, such as chemotherapy-resistant blastemal tumors, with a 5-years survival rate of 65%. Prognosis for WT patients is dependent on factors, such as age, tumor development stage, metastasis, and presence of diffuse anaplasia. Anaplasia refers to poorly or non-differentiated cells that can grow to about four times the size of healthy cells and also present hyperchromic and atypical mitosis (5). Although the molecular pathways involved in the pathogenesis of this tumor variant remain unclear, there is nevertheless some evidence indicating that microRNAs (miRNAs) contribute to the development of this neoplasia as epigenetic regulators and that miRNA expression differs between DAWT and FHWT variants (6).

MiRNAs are endogenous, non-coding RNAs, 18–21 nucleotides long (7). Their main functions are as post-transcriptional regulators of target mRNAs through inhibition of translation or mRNA degradation (8). MiRNAs were first identified as regulators of cellular differentiation, proliferation, and apoptosis. However, in recent years, they have also been shown to play important roles in tumorigenesis and pathogen–host interactions (9). Altered expression of certain miRNAs (called “oncomiRNAs”) has been associated with the development of specific tumors (10). Several studies have reported the dysregulation of miRNAs in various neoplasias, such as gastric cancer, thyroid cancer, and urothelial carcinoma, and the altered expression of these miRNAs has been proposed as diagnostic and prognostic biomarkers (11–14). Wilms tumor etiology includes dysregulated epithelial–mesenchymal transition (EMT), cell proliferation, invasion, cell migration, and metastatic potential. In the present study, we aimed to identify and describe dysregulated miRNA expression profiles in WT, based on clinicopathological characteristics of WT patients, primarily including the presence of anaplasia, metastasis, and association with the main observed genetic alterations. This allowed us, both to contribute to tumor biology and to propose potential prognosis biomarkers that can participate in tumor development.

## Materials and Methods

### Patients and Samples

Wilms tumor and non-neoplastic kidney tissue samples were collected at the Pathology Department of the Instituto Nacional de Salud, Hospital Infantil de México “Federico Gómez” (HIMFG) between 1994 and 2017. Approval was granted by the Bioethics Committee of HIMFG. To determine the global expression profile, a discovery cohort consisting of frozen tissue samples of four DAWT, eight FHWT, and six control tissues from pediatric patients' kidneys autopsy were used. A validation cohort consisted of frozen tissues and formalin-fixed, paraffin-embedded (FFPE) samples of 45 FHWT tissues, eight DAWT, and 17 controls were used.

### Sample Treatment

Formalin-fixed, paraffin-embedded tissue sections (FFPE) and frozen tissues derived from nephrectomies were used. Two pathologists confirmed the histopathological WT diagnosis and presence of tumor tissue with adequate morphology in 80% of the sample (to identify necrosis-free tissue samples). In total, 53 samples satisfying the above inclusion criteria were obtained and used for further analysis.

### RNA and DNA Isolation

For RNA and DNA isolation, tissues were digested with proteinase K (P-2308, Sigma-Aldrich, San Luis Missouri) at 42°C for 24 h. RNA was extracted using TRIzol reagent (15596018, Thermo Fisher Scientific, Waltham, Massachusetts), followed by DNAse treatment (AM1907, Thermo Fisher Scientific). DNA was extracted using phenol–chloroform–isoamyl alcohol (77617, Sigma-Aldrich). The quality and concentration of each RNA and DNA sample were verified using a NanoDrop ND-1000 (Thermo Fisher Scientific). Only samples with an A260/A280 ratio of 1.8–2.0 were analyzed.

### TaqMan Low-Density Arrays (TLDAs)

The discovery cohort consisting of four DAWT, eight FHWT, and six non-neoplastic control patient autopsy samples were included. All samples used in this phase were frozen tissue. cDNA was synthesized from 1 μg of total RNA using a Megaplex kit (4444745, Applied Biosystems). To create a miRNA expression profile, 756 miRNAs were divided into cardA v2.0 and B v3.0 and tested following the manufacturer's instructions. TaqMan MicroRNA Master Mix was used for amplification, performed in a Viia 7 thermocycler (Applied Biosystems). MiRNAs with Cq values >35 were considered non-informative and were excluded.

Tumoral Cq values were normalized to those of U6 snRNA and RNU48 endogenous controls and compared with those of non-neoplastic kidney tissues using the relative quantification formula 2^−ΔΔCt^. Heatmaps for the relative expression values (fold change) were generated using MultiExperiment Viewer (MeV) v.4.6. Data were evaluated by comparing expression values between DAWT, FHWT, and control samples. The SAM (Significance Analysis of Microarrays) program was used to evaluate significant differences between miRNAs groups. Values >2 were considered to correspond to upregulation, values < −2 to downregulation, and values between −2 and 2 to no change in expression.

### Quantitative Real-Time Polymerase Chain Reaction (RT-qPCR)

The cohort of validation consisting of 45 FHWT tissues and eight DAWT were tested by RT-qPCR. Based on the TLDA results, five miRNAs related to differentiation, EMT, and MET were chosen based on the importance of these in this type of tumor, as follows: miR-10a-5p, miR-29a-3p, miR-181a-5p, miR-200b-3p, and miR-218-5p. Three of these five miRNAs were then tested using *in situ* hybridization (ISH). cDNA was synthesized using the TaqMan MicroRNA Reverse Transcription kit (436696, Applied Biosystems), and 400 ng of the cDNA was used for qPCR. TaqMan probes (4427975, Applied Biosystems) were tested for each miRNA (miR-10a-5p [ID 000387], miR-29a-3p [ID 002112], miR-181a-5p [ID 000480], miR-200b-3p [ID 002251], and miR-218-5p [ID 000521]) to obtain relative quantification. Tumoral Cq values were normalized to those of U6 snRNA [ID 001973] endogenous control and compared with those of non-neoplastic kidney tissues using the relative quantification formula 2^−ΔCt^. Expression values for each miRNA were averaged in each tumor type and healthy kidney tissue. In addition, the number of cases of each tumor type with high and low expression was determined. Analysis of qPCR data was performed using the Kruskal–Wallis test and *post-hoc* Dunn's test in GraphPad Prism v.8.0 (GraphPad Software, La Jolla, CA, USA). *P* < 0.05 was considered significant.

### *In silico* Analysis

Molecular interactions in the biological process for the 109 significant miRNAs obtained from TLDA were searched through the DIANA TOOLS database (15), and those for the five analyzed miRNAs were separated. These miRNAs were mapped to target genes using the miRNET database (16) and genes or transcription factors that regulate these five miRNAs were determined through TransmiR database v2.0 (17).

### *In situ* Hybridization

Locked nucleic acid (LNA) probes (339111, Qiagen, Hilden, Germany) were used for ISH for three miRNAs: miR-181a (ID 00612607), miR-200b (ID 00619853), and miR-218 (ID 00610912), in 17 FHWT, five DAWT, and 22 control tissue samples (residual tissue adjacent to the tumor). FFPE slices (5 μm) were used in electro-charged slides (71864-01, SuperFrost Plus Gold Slide, Hatfield, PA). Samples were deparaffinized by drying in a conventional oven for 45 min at 60°C 24 h before assay. ISH was performed using a miRCURY LNA miRNA ISH Optimization Kit (FFPE) (339111, Qiagen) following the manufacturer's instructions, with some modifications. Briefly, the hybridization temperature was increased from 55 to 56.8°C (hybridization oven, UVP, HB-1000 Hybridizer, Hampton, New Hampshire), and the washing steps were performed with saline sodium citrate (SSC) solutions as follows: two washes with 5 × SSC, one wash with 1 × SSC, and two washes with 0.2 × SSC. All the washing steps were performed at the hybridization temperature, except for the last wash (0.2 × SSC), which was performed at room temperature. A positive control (miR-126), negative control (scramble sequence), and endogenous control (U6sn) (339455, Qiagen) were also used.

Digital images of the tissues were obtained at ×40 magnifications, and miRNA abundance was quantified by total pixel (TP) and nuclear (N) count using the Aperio ImageScope system (Leica Biosystems, Wetzlar, Germany). Low-, medium-, and high-intensity values were first obtained, and these values were then divided by the analyzed area and summed to obtain the total intensity in tumoral and residual tissues. Data analysis was performed using the Kruskal–Wallis test in GraphPad Prism v8.0; *P* < 0.05 was considered significant.

### Multiplex Ligation-Dependent Probe Amplification (MLPA)

Relative DNA copy number variation (CNV) was determined using semiquantitative MLPA. A total of 19 samples were treated, using 250 ng of DNA per sample. Initially, DNA was denatured for five min at 98°C. Then, hybridization, ligation, and amplification with end-point PCR were performed using the SALSA MLPA P380-A1 Wilms Tumor probe mix kit following the manufacturer's instructions. Reactions were performed in an MJ Mini Personal Thermal Cycler (Bio-Rad, Hercules, Cal). Finally, capillary electrophoresis was carried out in an ABI-3730XL (Thermo Fisher Scientific, Hampton, Hampshire). The obtained results were analyzed with Coffalyyser.Net.Software (MRC Holland, Amsterdam, NLD). Only deletions with values <0.7 and duplications with values >1.2 were considered. This test includes up to three probes for some genes; thus, for the analysis we consider at least two altered probes in these genes.

### Statistical Considerations

For statistical analyzes, group data were analyzed using the Kolmogorov–Smirnov (KS) normality test. No normal distribution data were found; also, Brown–Forsythe and Bartlett's homoscedasticity tests were performed for homogeneity of variance between group data. These tests showed non-comparability behavior, so non-parametric tests were performed. Statistically significant differences between miRNA expression and clinical characteristics, such as age, gender, pre-surgery treatment, unfavorable histology cases, and metastasis cases were analyzed using the Kruskal–Wallis test (mean rank) with Dunn's multiple-comparison *post-hoc* test. At last, for determining if miRNA expression and case distribution can discriminate between favorable histology and metastasis cases, we applied the receiver operating characteristic (ROC) curve and predictive precision was determined by measuring the area under the curve (AUC), sensibility, and specificity.

## Results

### Clinicopathological Characteristics

Clinicopathological data were obtained from patients to correlate molecular assays as described in Table 1. A total of 53 samples were included, and information was absent for only one patient. Patients had an average age of 43 months (~3.5 years). In addition, the lung was the most common site of metastasis (41.5%). Stage III was the most frequent (28.3%). Preoperative treatment was given to only 38.3% of patients. Finally, DAWT cases accounted for only 15.1% (8/53) of the total WT samples evaluated.

**Table 1:** Clinicopathological data of Wilms tumor patients.

**Clinical parameters**	**Description**	**# Cases**
Age	Mean = 43 months	
Gender	Female	31
	Male	22
Laterality	Left	20
	Right	29
	Bilateral	4
Tumor histology	Favorable histology	45
	Unfavorable histology (anaplastic)	8
Stage	I	7
	II	13
	III	15
	IV	13
	V	4
Treatment	Preoperative chemotherapy	22
Metastasis	Lung	12
	Lymph nodes	3
	Liver	2
	Peritoneum	3
	Kidney	1

### TLDA

**Figure 1:**
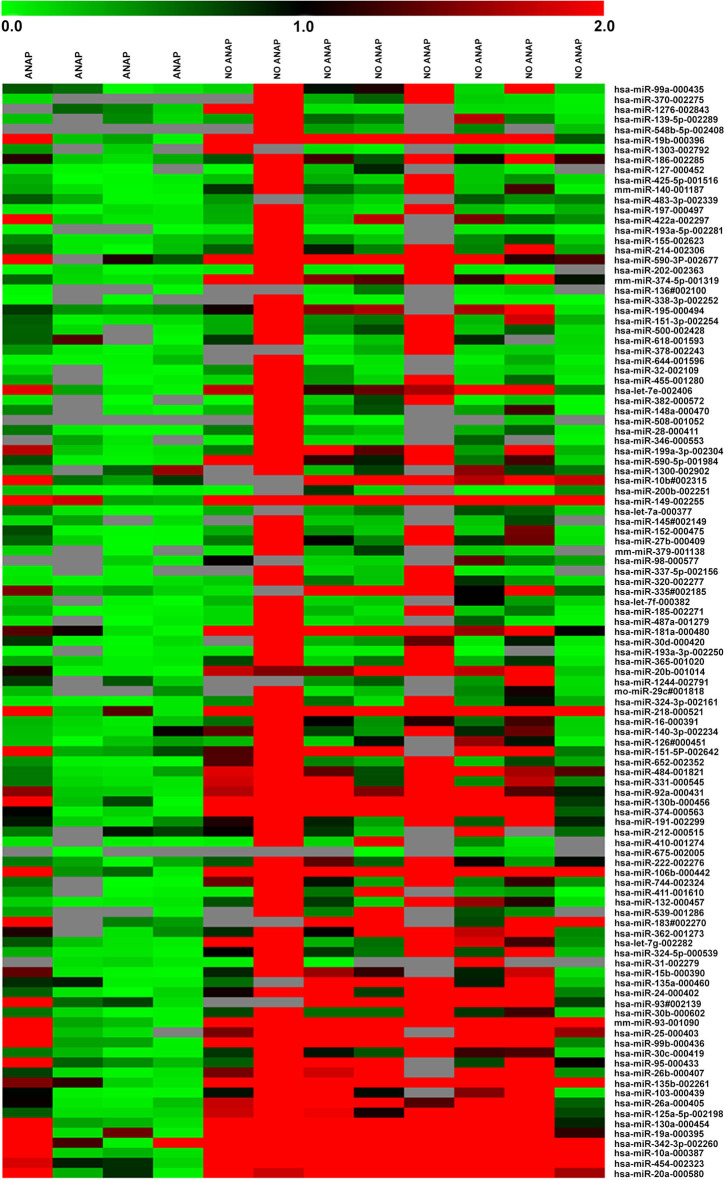
Heat map showing the significant relative expression of microRNAs in four DAWT and eight FHWT samples based on TaqMan low-density array (TLDA). Red indicates increased expression (values >2), black indicates no change in expression (values between −2 and 2), and green indicates reduced expression (values < −2). The map was generated using MeV software.

For miRNA expression profiles, 756 miRNAs were evaluated per sample. A total of 220 miRNA amplicons were obtained (Supplementary Figure 1). Amplicons were analyzed through SAM and a total of 109 (48.7%) miRNAs differentially expressed between DAWT and FHWT samples were identified (Figure 1). A group of miRNAs was selected based on differential expression, mainly between DAWT and FHWT samples. Upregulated and downregulated miRNAs were chosen and validated in the next stage (miR-10a, miR-29a, miR-181a, miR-200b, and miR-218). MiR-10a, miR-181a, and miR-218 showed the highest expression levels in FHWT tissues, while miR-29a and miR-200b presented reduced expression in both types of WT tissue, although expression was lower in DAWT.

### RT-qPCR

**Figure 2:**
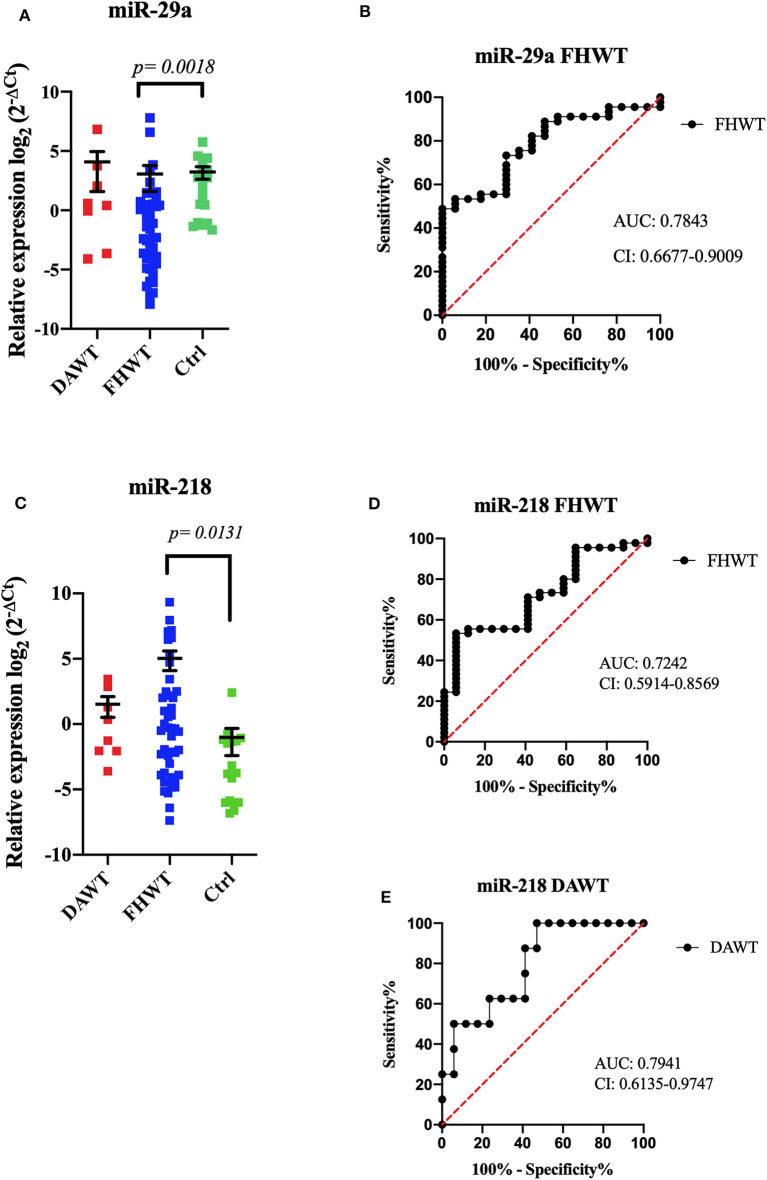
Relative expression of microRNAs by RT-qPCR. The dot plot shows the relative expression of miRNAs in DAWT (*n* = 8), FHWT (*n* = 45), and controls (*n* = 17). MiR-29a showed a significant difference between FHWTs and Ctrls (*p* = 0.0018, mean ± SEM) **(A)**. ROC curve analysis demonstrated that miR-29a could distinguish patients with FHWTs from controls **(B)**. MiR-218 showed significant differences between FHWTs and Ctrls (*p* = 0.0131, mean ± SEM) **(C)**. ROC curve analysis could discriminate against patients with FHWTs from controls **(D)** and DAWT from controls **(E)**. Kruskal–Wallis tests with Dunn *post-hoc* were performed using *p* < 0.05. AUC, area under the curve; CI, confidence interval.

MiRNA relative expression levels and its comparisons with DAWT (*n* = 8), FHWT (*n* = 45), and control group (*n* = 17) showed statistically differences. MiR-29a showed lower expression levels in FHWT compared to the control group (*p* = 0.0018), and miR-218 showed higher expression levels in FHWT than controls (*p* = 0.0131); DAWT comparisons did not show significant differences (Figure 2). ROC curves were obtained too for discriminating between miRNA expression and FHWT. MiR-29a AUC was 0.7843 (sensibility 73%, specificity 70.5%). For miR-218, AUC was 0.7242 (sensibility 71%, specificity 59%); although significant statistical differences were not found between DAWT and the control group, ROC curve showed high levels (AUC = 0.7941, sensibility 62.5%, specificity 76%) (Figure 2). The remaining three miRNAs did not show significant differences between any evaluated groups (Supplementary Figure 2).

**Figure 3:**
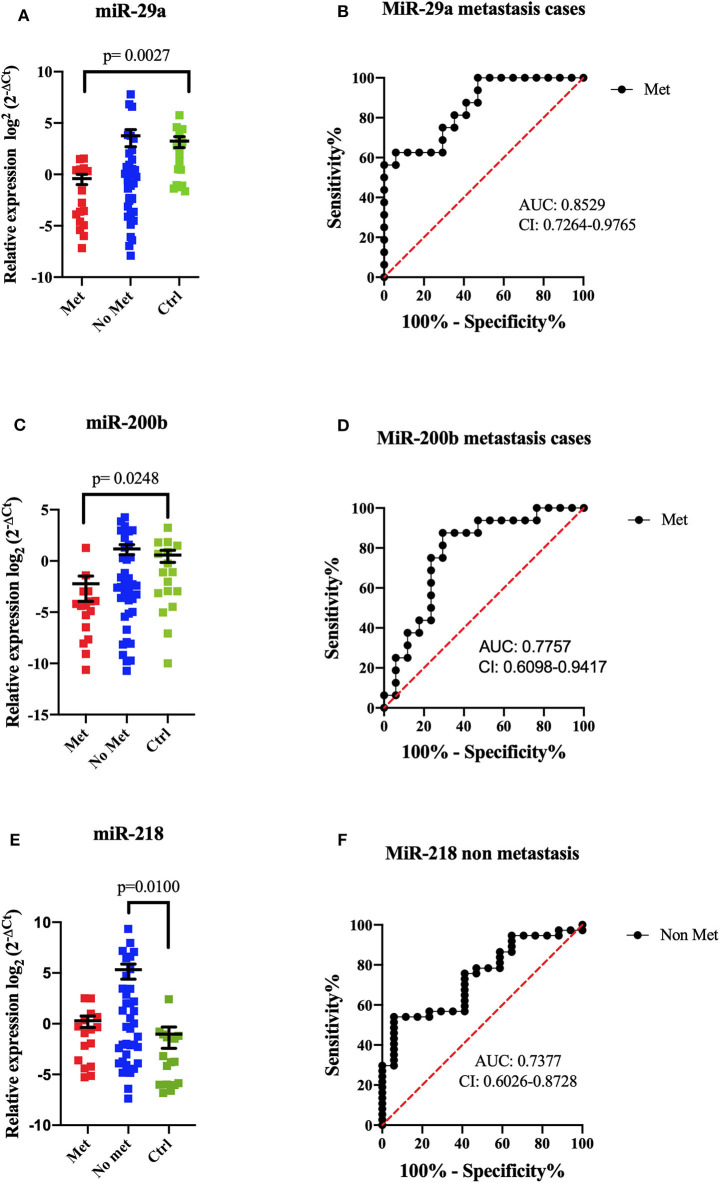
MiRNA expression comparison between metastatic and non-metastatic patients by RT-qPCR. The dot plot shows the relative expression of miRNAs in metastatic tumors (Met, *n* = 16), non-metastatic (No Met, *n* = 37), and controls (Ctrl, *n* = 17). MiR-29a showed a significant difference between metastatic tumors and controls (*p* = 0.0027, mean ± SEM) **(A)**. ROC curve analysis demonstrated that miR-29a could distinguish patients with metastatic from controls **(B)**. MiR-200b showed a significant difference between metastatic tumors and controls (*p* = 0.0248, mean ± SEM) **(C)**. ROC curve analysis could discriminate against patients with metastasis from controls **(D)**. MiR-218 showed significant differences between non-metastatic tumors and controls (*p* = 0.0100, mean ± SEM) **(E)**. ROC curve analysis could discriminate against patients without metastasis from controls **(F)**. Kruskal–Wallis tests with Dunn *post-hoc* were performed using *p* < 0.05. AUC, area under the curve; CI, confidence interval.

A comparison between metastatic (*n* = 16), non-metastatic (*n* = 37), and control groups (*n* = 17) was done for expression levels; miR-29a showed an important decreased expression (*p* = 0.0027) compared to the control group, and the ROC curve discriminated metastatic patients with high specificity and sensibility (AUC = 0.8529, sensibility 75%, and specificity 70%) (Figure 3). In this tumor group, miR-200b also showed lower levels in metastatic patients (*p* = 0.0248) than the control group (AUC = 0.7757, sensibility 75%, and specificity 77%). Both miRNAs allowed to discern between metastatic patients and control groups; nevertheless, significant differences were not shown compared to non-metastatic patients groups (Figure 3). In non-metastatic cases, higher expression levels of miR-218 were obtained than the control group (*p* = 0.0100). The ROC curve showed an AUC value = 0.7377, sensibility 75%, and specificity 59% (Figure 3).

Another evaluated clinic factor was the miRNA expression level difference between pre-surgery treatment cases (*n* = 22), without pre-surgery treatment (18), and control groups (*n* = 17). About this, miR-218 showed higher expression levels in non-pretreated patients than the control group (*p* = 0.0220); also, miR-29a showed lower expression levels than the control (*p* = 0.0074). No significant differences were shown between pretreated and non-pretreated patient groups. These results showed that miRNAs levels were not affected by pre-surgery treatment, and it suggests that expression changes are due to tumor development (Supplementary Figure 3).

Gender comparisons showed that miR-181a and miR-218 had significant differences compared between male and control groups with values of *p* = 0.0255 and *p* = 0.0005, respectively, obtaining higher expression levels on male patients; also, miR-29a showed differences but compared to female ones (*p* = 0.0016). In this case, females showed lower expression levels than controls did (Supplementary Figure 4). Referring to expression level comparison among the five WT tumor stages, only miR-29a showed differences between stage III and the control group (*p* = 0.0335), which had higher expression levels than those of stage III (Supplementary Figure 5). For the age ranges, only two miRNAs showed statistically significant differences: miR-29a comparison between the control group and the 0–1.9-years-old range (*p* = 0.0456), obtaining higher expression levels in the control group, and miR-218 comparison between control group and the 2–3.9-years-old range (*p* = 0.0492) with lower expression levels in the control group (Supplementary Figure 6).

Finally, comparing between the types of two more frequent histological tissues (blastemal and triphasic) and controls, a significant difference between blastemal tissue and control group was showed in miR-29a (*p* = 0.0136) and miR-218 (*p* = 0.0099), whereas triphasic tissues had differences compared to control only in miR-29a expression levels (*p* = 0.0155). MiR-29a showed higher expression levels in controls, whereas in the miR-218 control group expression levels are lower (Supplementary Figure 7).

### *In silico* Analyses

We used the DIANA TOOLS database to detail the biological pathways associated with the 109 differentially expressed miRNAs (Supplementary Figure 8). The seven main pathways identified were the p53 signaling pathway, viral carcinogenesis, cell cycle, hepatitis B, prostate cancer, cancer pathways, and bladder cancer.

**Figure 4:**
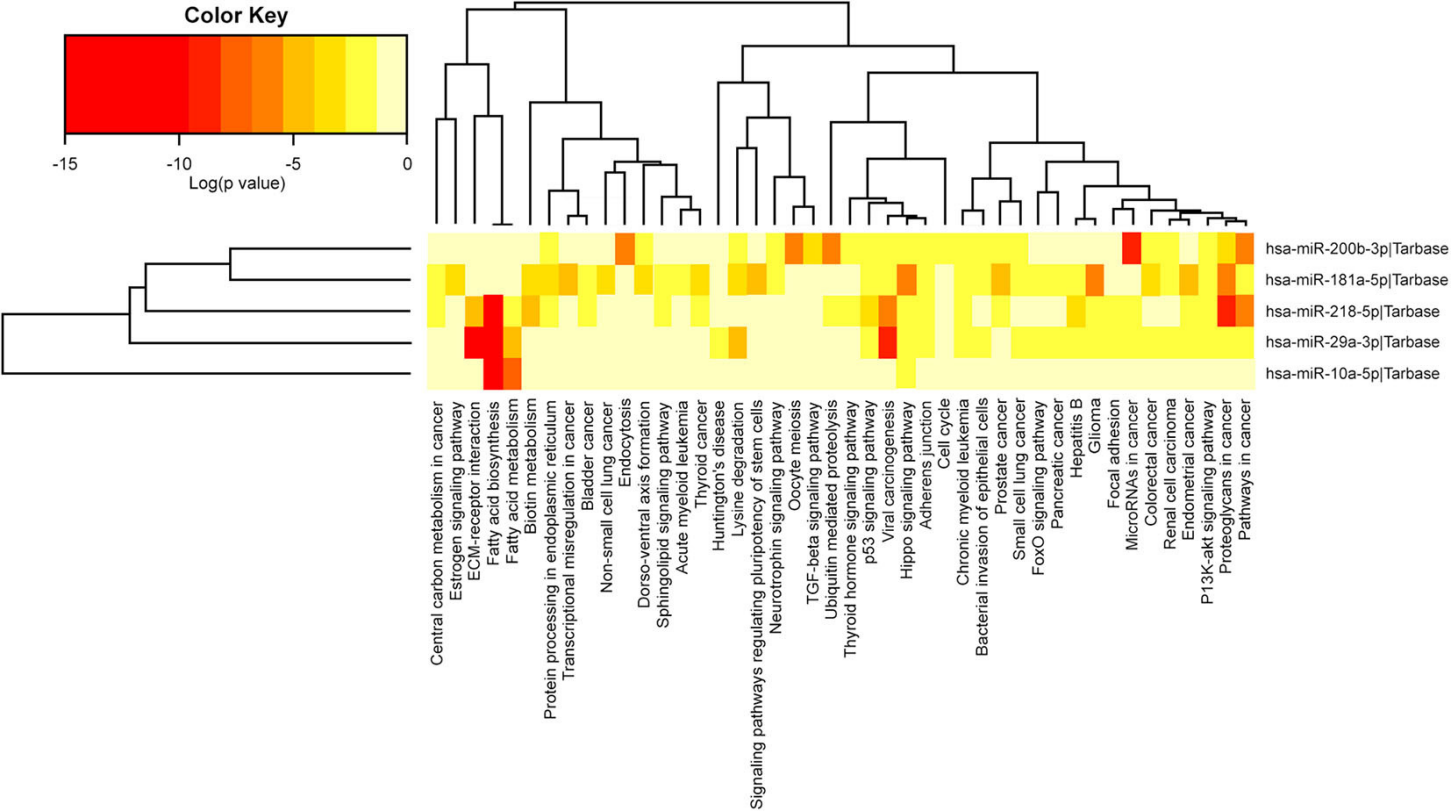
Biological pathways related to the 5 miRNAs selected by RT-qPCR through the miRPath v.3 database (DIANA TOOLS). Red indicates the pathways where miRNAs have low significant values, and beige indicates no difference. In both dendrograms, the *x*-axis labels show hierarchical groupings corresponding to the pathways and the *y*-axis labels hierarchical groupings corresponding to miRNAs. The five miRNAs are involved in one common pathway, the Hippo signaling pathway, while four (not miR-10a) are involved in the following nine routes: cancer routes, proteoglycans in cancer, PI3K/Akt signaling pathway, chronic myeloid leukemia, adherens junctions, viral carcinogenesis, p53 signaling pathway, fatty acid biosynthesis, and fatty acid metabolism.

In the case of the 5 selected miRNAs, we observed them to participate in the Hippo signaling pathway, involved in the inhibition of tumor suppressor genes (19). Four miRNAs, namely, miR-29a, miR-181a, miR-200b, and miR-218, participated in the pathways: cancer routes, proteoglycans in cancer, PI3K/Akt signaling pathway, chronic myeloid leukemia, adherent junctions, viral carcinogenesis, p53 signaling pathway, fatty acid biosynthesis, and fatty acid metabolism (Figure 4).

We used the miRNet database to identify target genes for the analyzed miRNAs. We found that *MYCN* and *WTX* (*AMER 1*) are miR-29a target genes, while MYCN is also a target for miR-200b; *IGF2* and *TP53* are targets for miR-218. Moreover, TransmiR v.2.0 database analysis indicated that several transcription factors that regulate miRNAs, such as *MYCN*, also promote miR-181a and miR-218 transcription. *MYCN* activates miR-10a and suppresses miR-200b, while TP53 activates miR-10a and miR-200b but suppresses miR-29a.

### *In situ* Hybridization

**Figure 5:**
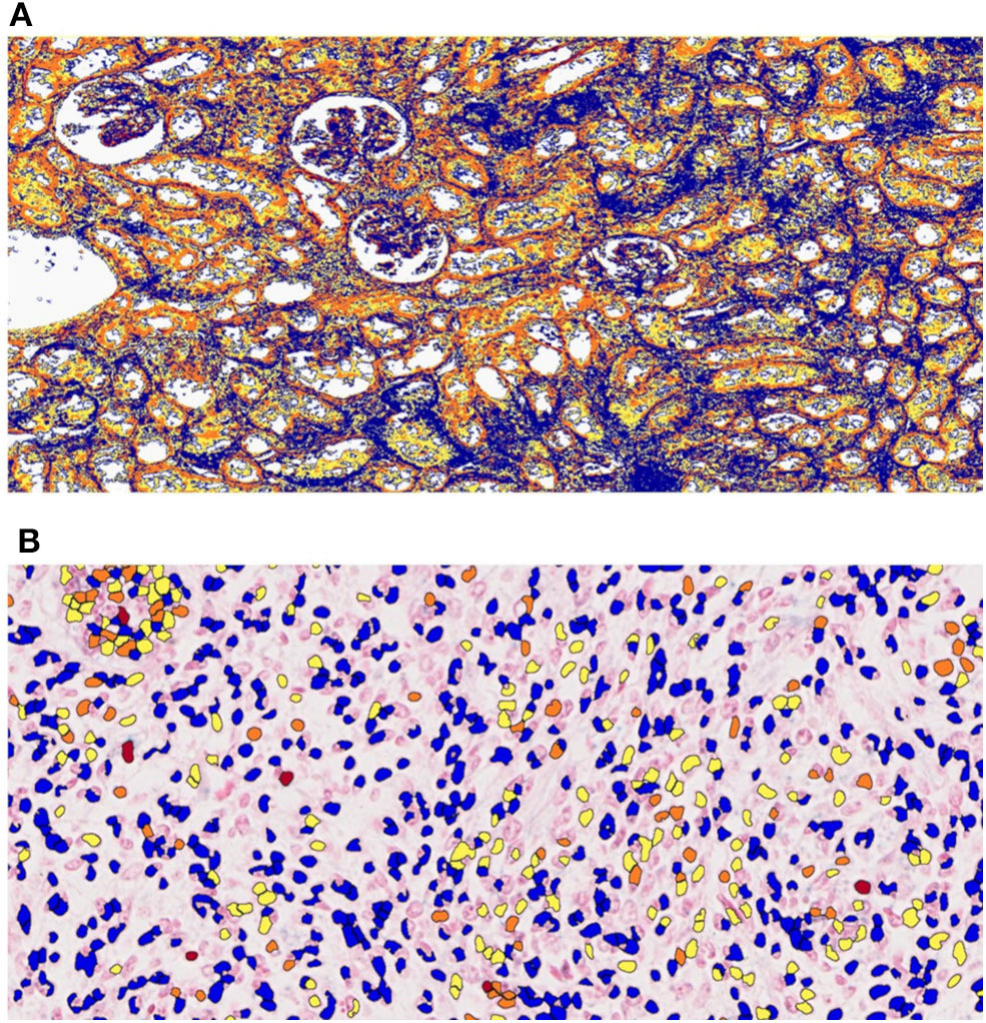
Analysis of *in situ* hybridization quantification data using the Aperio ImageScope system. ImageScope software was used to quantify **(A)** total pixel (TP) and **(B)** nuclear (N) staining intensity values. Red represents positive, orange represents moderately positive, yellow represents slightly positive, and blue represents negative zones or nuclei.

**Figure 6:**
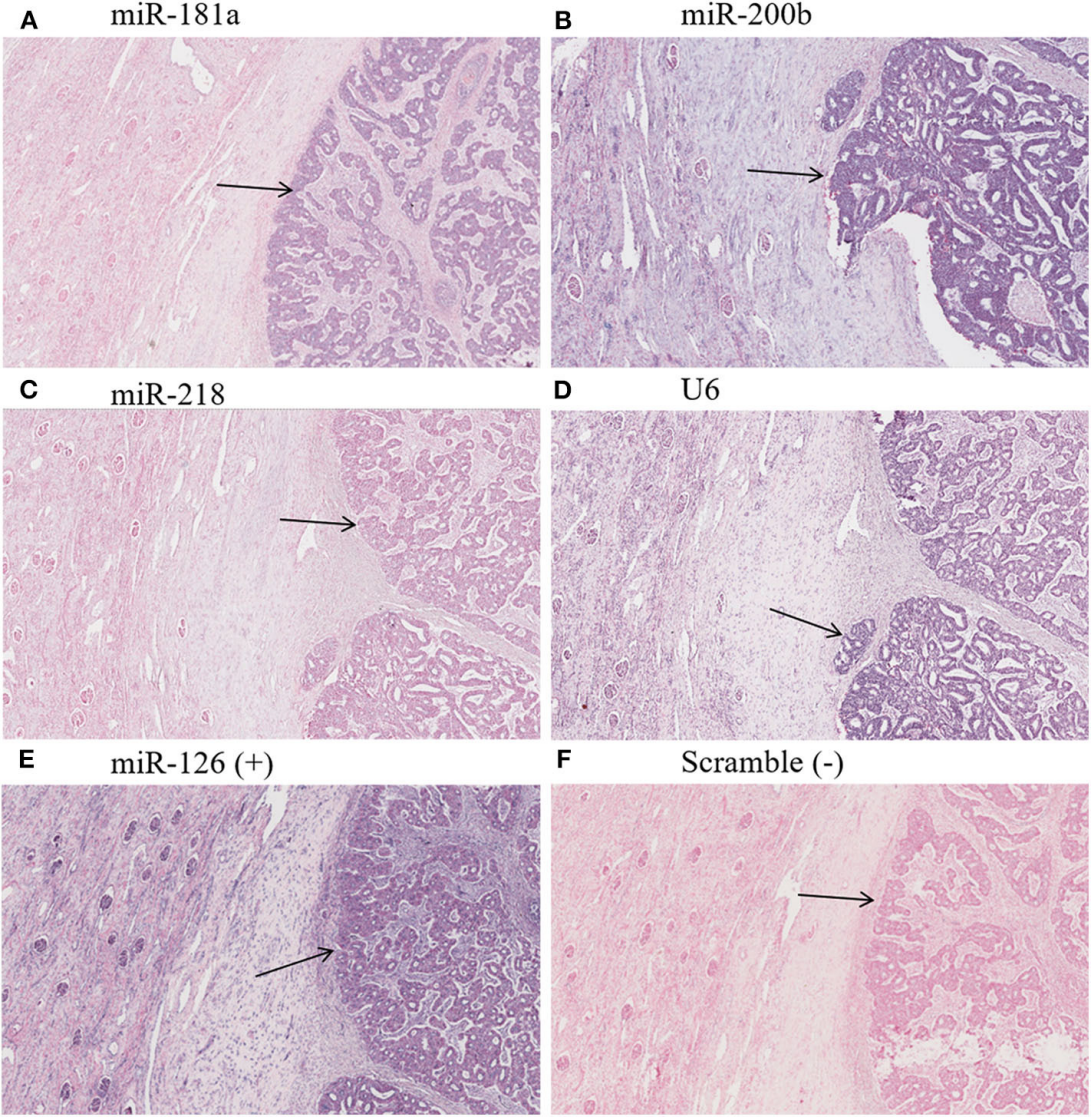
Hybridization *in situ* with an FHWT LNA probe. Arrows indicate tumor tissue evaluated with probes to miR-181a **(A)**, miR-200b **(B)**, miR-218 **(C)**, U6sn endogenous control **(D)**, positive control miR-126 **(E)**, and negative control scramble **(F)**. Purple color shows respective evaluated miRNA-positive cells, whereas red/pink color shows negative cells. **(A–C)** Demonstrate increased miRNA in comparison to adjacent tissue. 20× magnification picture obtained from ImageScope software.

**Figure 7:**
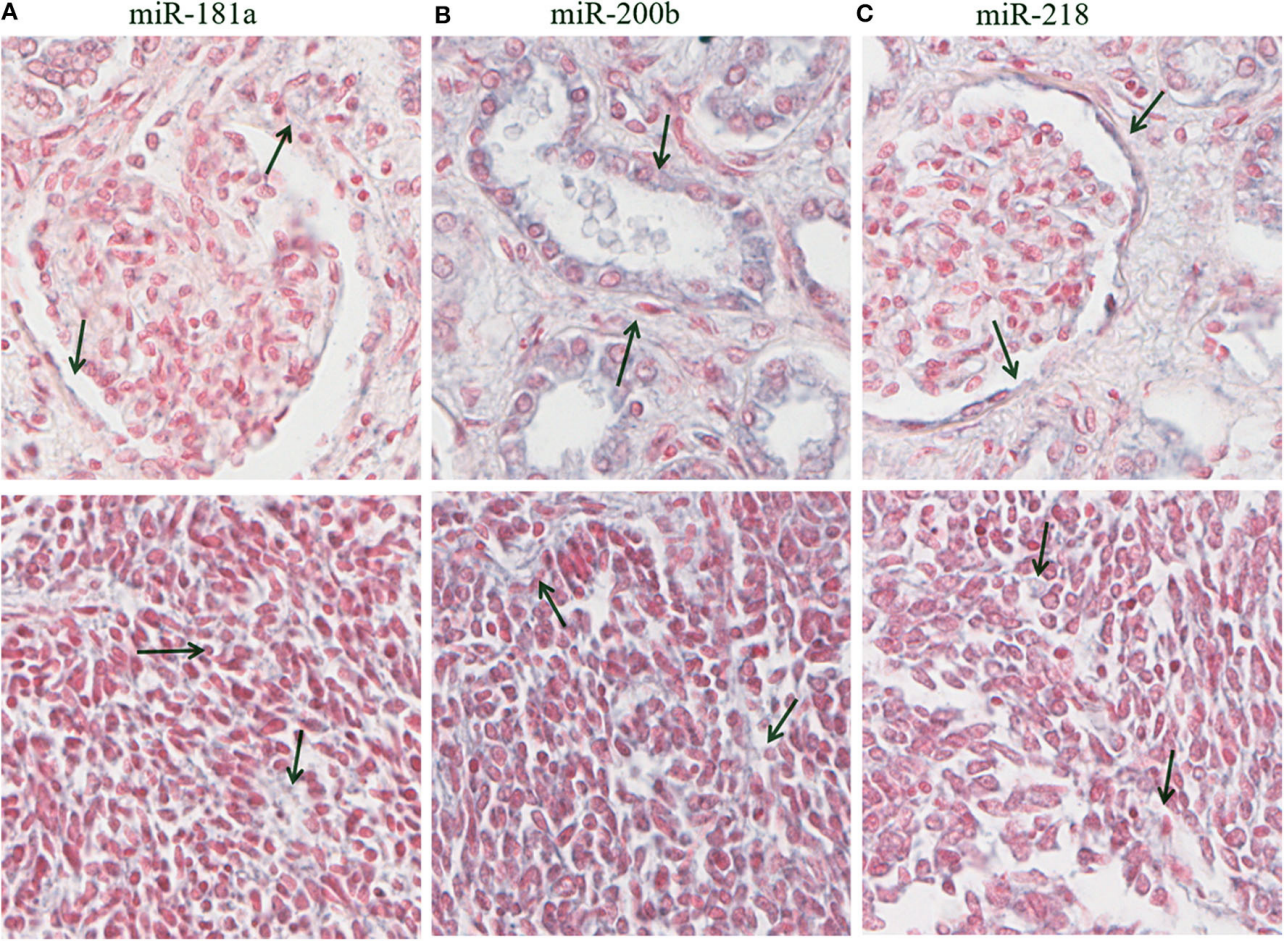
Hybridization *in situ* in residual and tumor tissues. In the top row are observed healthy kidney tissues, whereas in the bottom row are observed FHTW tumoral tissue blastemal cells corresponding to miR-181a **(A)**, miR-200b **(B)**, and miR-218 **(C)**. Purple zones are indicated with arrows corresponding to positive zones in healthy tissue. 16× magnification picture obtained from Aperio Image Scope.

**Figure 8:**
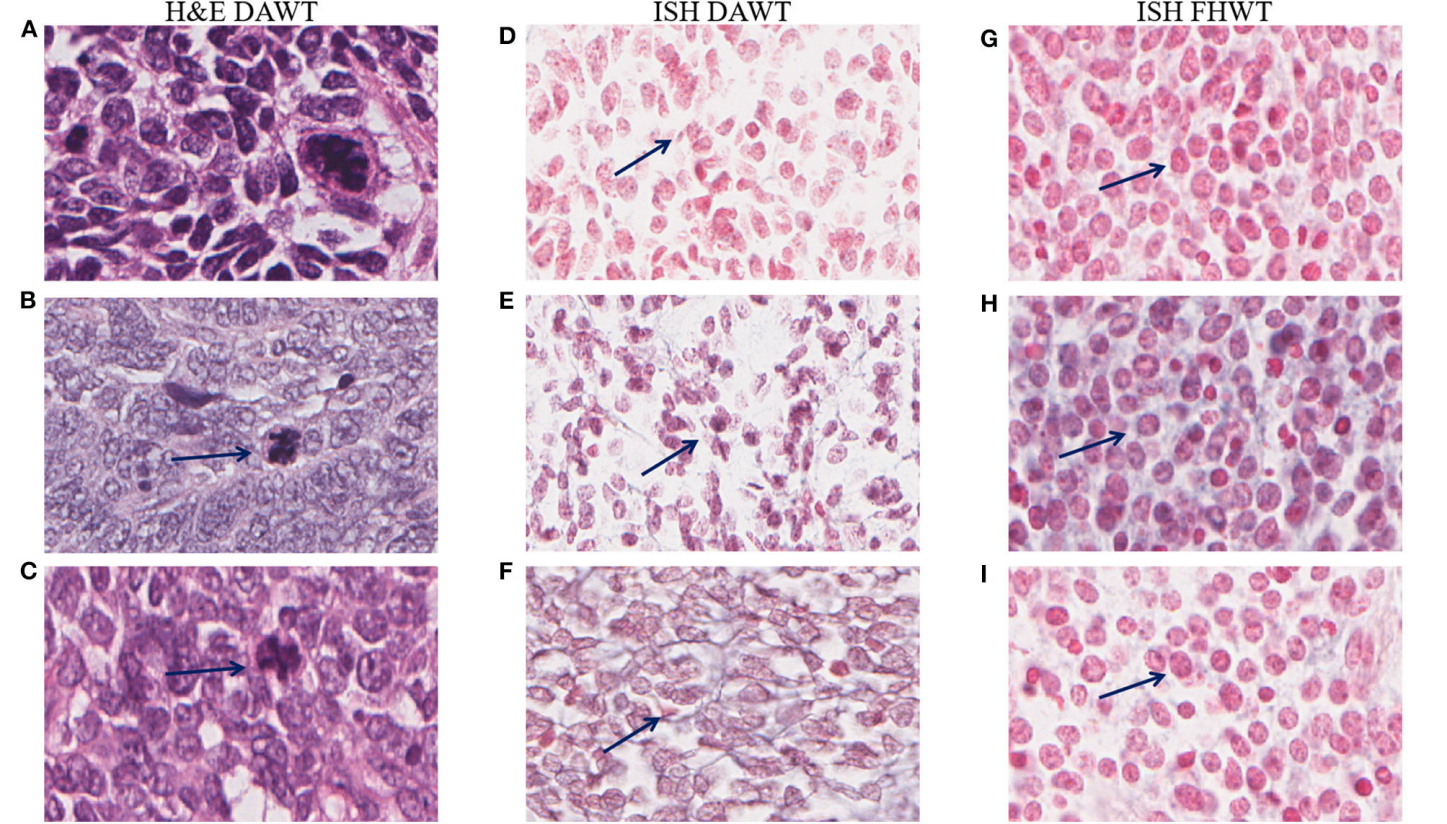
Comparison of DAWT and FHWT *in situ* hybridization (ISH) data, and representative images of anaplastic cells. The first column shows anaplastic cells **(A–C)** stained with hematoxylin and eosin (H&E) corresponding to each case in the second column. Anaplastic cells are marked with arrows in the second column, as their appearance is not clearly indicated by ISH. Comparison of DAWT (second column) and FHWT (third column) in two tumors with blastemal morphology. **(D,G)** miR-181a, **(E,H)** miR-200b, and **(F,I)** miR-218 showing higher staining intensities in FHWT tissues than in DAWT tissues; some positive cells are indicated with arrows. Pictures were obtained with an Aperio Image Scope at ×28.8 magnification.

Optical microscopy analysis of N and TP *in situ* hybridization (Figure 5) showed that staining intensity was greater in tumor tissue than in healthy kidney tissue (Figures 6, 7). Hybridization differences were determined between each of the three miRNAs for both FHWT (*n* = 17) and DAWT (*n* = 5) tissue samples (Figure 8).

**Figure 9:**
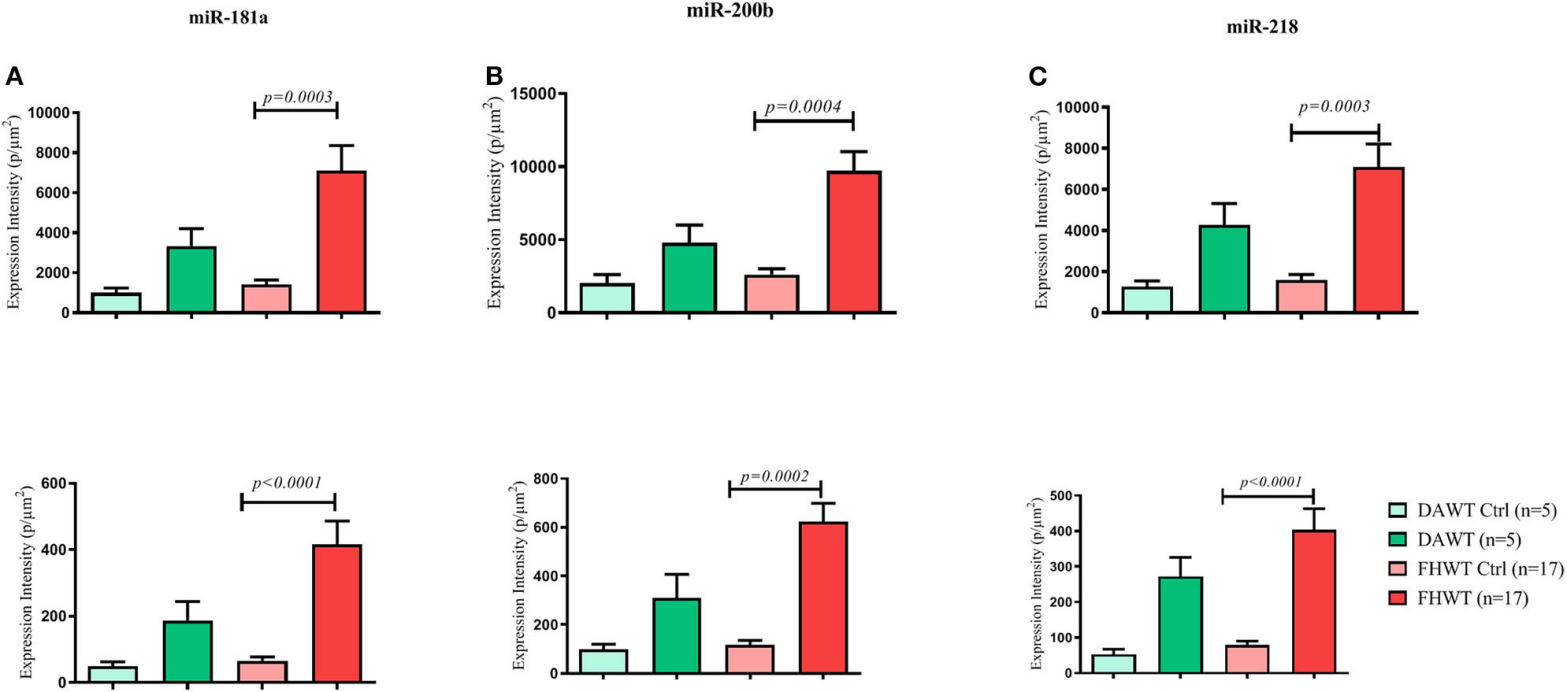
Comparison of miRNA expression in DAWT tissue, FHWT tissue, and the respective control groups (adjacent non-neoplastic kidney tissue). Expression comparison of miR-181 **(A)**, miR-200b **(B)**, and miR-218 **(C)** was performed through two analyses: top row: Total Pixels (TP), and bottom row: Nuclear (N). All comparisons showed significant differences between FHWT and their respective control group. Kruskal–Wallis tests with Dunn's *post-hoc* were performed using *p* < 0.05.

N and TP intensity values were normalized by their area (μm^2^) and grouped for the comparison between the two WT types, and both analyses were performed for miR-181a, miR-200b, and miR-218. Both analysis types over the three evaluated miRNAs showed significant differences only between FHWT and its respective control groups, in which FHWT tumor tissue expression levels are higher. For TP analysis, results were obtained as follows: miR-181a (*p* = 0.0003), miR-200b (*p* = 0.0004), and miR-218 (*p* = 0.0003), whereas N analysis showed these *p*-values: miR-181a (*p* < 0.0001), miR-200b (*p* = 0.0002), and miR-218 (*p* < 0.0001). To determine differences between groups, Kruskal–Wallis test and Dunn's multiple comparisons *post-hoc* were performed (Figure 9).

Also metastatic (*n* = 6) and non-metastatic (*n* = 15) cases were evaluated through this technique. PT analysis showed significant differences between the non-metastatic group and its adjacent healthy tissue with values of *p* = 0.0020 for miR-181a, *p* = 0.0023 for miR-200b, and *p* = 0.0004 for miR-218, the same for N analysis but with values of *p* = 0.0001 for miR-181a, *p* = 0.0006 for miR-200b, and *p* < 0.0001 for miR-218. The non-metastatic group showed higher expression than did healthy tissues. Altogether, ISH assay showed an increase in tumor miRNA expression (Supplementary Figure 9).

ISH staining intensity was also evaluated for the three cell types in FHWT triphasic tumors (*n* = 11), but no significant difference was found in the levels of any of the miRNAs tested; however, all three cell types showed a similar tendency, with mesenchymal cells exhibiting the lowest expression and epithelial cells the highest expression (Supplementary Figure 10).

### MLPA

The MLPA assays showed genetic alterations in 19 WT patients. Changes in gene copy number can influence patient prognosis. The most frequently observed alterations were the *WTX* deletion/1q duplication, recorded in 5 cases (26%); *WTX* deletion/16q deletion, observed in 3 cases (16%); and 1q duplication/16q deletion, recorded in 2 cases (10%). Deletion of the 1p36 chromosomal locus was detected in 3 cases (16%). The *WTX* gene was altered in 13 cases (68%), representing 9 deletions (47%) and 4 duplications (21%). Gain of 1q was observed in 7 cases (37%) and was one of the most constant alterations recorded. The *WT1* gene was altered in 5 cases (26%), while *IGF2* was deleted in 4 cases (21%). There was an increase in the *MYCN* gene copy number in 1 case (5%), and deletion of this gene was found in 3 cases (15%); deletion of the *TP53* gene was found in 4 cases (21%) (Table 2). Analysis of miRNA chromosomal loci further showed that the 1p36.33 locus, comprising miR-200b-3p, was deleted in three cases (16%).

**Table 2:**
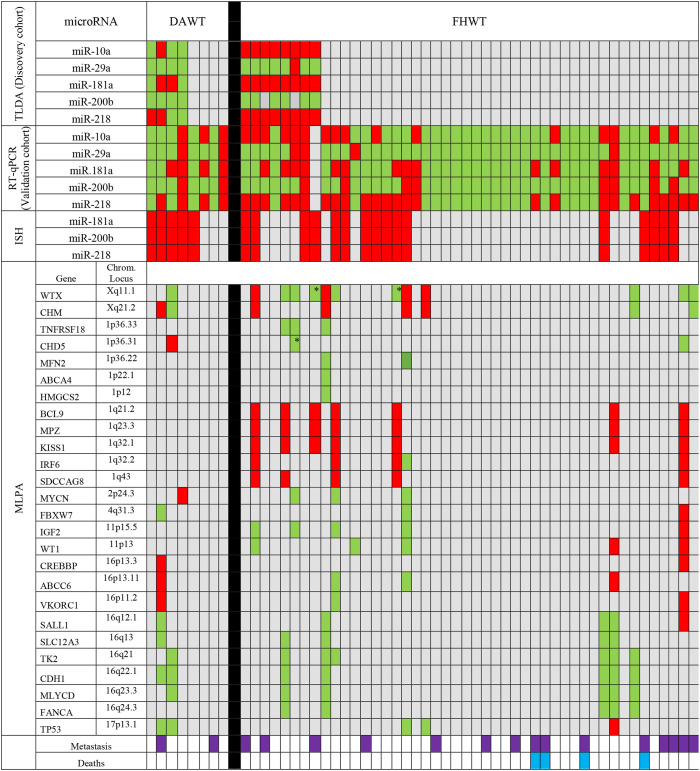
Summary results for all different WT tests and assays.

*Red color indicates overexpression, green color indicates low expression, and gray color indicates null expression. The purple color was assigned to metastatic cases and blue color was assigned to cases of people who died*.

In the MLPA, the green areas show relative probe signals with values <0.7 (deletion/partial deletion); the red areas show relative probe signals with values >1.2 (duplication/partial duplication). ^*^Homozygous deletion.

*TLDA: DAWT n = 4 y FHWT = 8; RT-qPCR DAWT n = 8 y FHWT n = 45; ISH DAWT n =5 y FHWT n = 17*.

## Discussion

Wilms tumor presents a high degree of genetic and epigenetic heterogeneity. Although structural alterations in numerous genes have been associated with WTs, several cases have been identified where no mutations are present, suggesting that other mechanisms may be involved in WT etiology (20). MiRNAs are known to play roles in the epigenetic mechanisms involved in tumor development and are involved in numerous cellular pathways through their interactions with a wide variety of transcription factors. In this study, we evaluated the role of miRNAs in WTs according to favorable or unfavorable histology and considering clinicopathologic variables. The expression of the evaluated miRNAs in DAWT and FHWT samples showed changes between the two tumor types. The miRNAs analyzed in this study were previously reported to be related to tumor development through the impairment of important molecular pathways (21).

In this study, differential expression of miR-10a, miR-29a, miR-181a, miR-200b, and miR-218 was found through TLDA assay; nevertheless, there should be a greater emphasis in miR-29a, miR-200b, and miR-218 because of these showing results on validation cohort trials (RT-qPCR and ISH). In the context of miR-29a-5p, lower expression levels in FHWT compared to the control group were shown, whereas in metastatic comparison, these miRNA expression levels were evidently lower compared to the control group as well. The results obtained together showed that miR-29a decreases in tumor tissues compared to healthy kidney tissue; it also decreased in tumors with favorable histology and metastatic tumors, consistent with the gastric cancer data reported by Zhao et al. in 2018 (22). In metastatic prostate cancer, its expression also diminished and it was demonstrated that it participates in cell proliferation inhibition (23). Nevertheless, this miRNA does not participate similarly in all cancer types; Pei et al. in 2016 observed that this miRNA expression was upregulated and it favors breast cancer progression (24).

In our study, miR-29a lower expression was emphasized in metastatic developed tumor cases, so this could mean that its inhibitor function was affected in these groups. On the other hand, this miRNA was involved in EMT promotion when it was overexpressed (25); in this case, our study showed no differences between FHWT and DAWT cases, whereby this activity could not be particularly related to FHWT or DAWT. In summary, miR-29a may serve different functions depending on the studied cancer type, and its expression is likely to be associated with different cellular mechanisms. The AUC obtained from ROC curves showed that it could be a marker that separates the more unfavorable development cases and this diminishing origin must be studied through its related genes and globally with its altered molecular pathways. This miRNA has not been elucidated previously on WT.

In the context of miR-200b-3p, in metastatic tumors TLDA and RT-qPCR techniques showed a statistically significant lower expression than the control group, so their expression decreased in this unfavorable prognostic group. However, ISH showed contrary results. These differences may be due to the following factors: (a) in the amplification techniques, a much bigger population of cells (three sections of 10 μm) was used, whereas in ISH the population is smaller (1 cut of 5 μm); (b) in ISH, a smaller number of cases (6 cases) were evaluated; and (c) ISH is considered a semiquantitative technique and its main objective was only to support the location and distribution of the miRNA. The miR-200b family is involved in diverse processes, such as the inhibition of metastasis, EMT, and angiogenesis (26), thereby contributing to a disordered cellular microenvironment in anaplastic processes. The expression of this miRNA has been reported to be downregulated in WT, affecting EMT, altering epithelial differentiation, and promoting the predominance of undifferentiated cells (27). Similarly, Braun et al. showed that the expression of miR-200 family members was reduced in anaplastic thyroid cancer compared with that in follicular thyroid cancer (28). Interestingly, the effects of this miRNA may be dependent on tumor type, as its expression has been reported below in lung, pancreas, colon, and breast cancers, but high in non-small cell lung and endometrial cancers (18, 29). Both for FHWT comparisons with the control group and with the metastatic group, ROC curve AUC was determined, where similarly to miR-29a, miR-200b is considered to be a marker for separation of more unfavorable development cases.

We found that miR-218-5p expression was higher in the two tumor types than in the controls but significantly different from FHWT. Corroborating these results, also the miR-218 metastatic comparison, differently from miR-29a and miR-200b, presents differences between non-metastatic and control groups with a higher expression presented by the non-metastatic group. Again, the AUC value obtained from ROC curves suggests that this miRNA could be a marker in tumors with favorable prognosis. It is important to consider that this miRNA is transcribed at two loci, such as miR-218-1 located on chromosome 4p15.31 and miR-218-2 on 5q34, which can influence the abundance (30). The role of miR-218 in WT is unknown. In WT, there are few studies that have reported it; one of them evaluated its expression in eight serum samples from WT patients and in eight predominantly blastemal FHWT tumors, and in both cases, no changes in its expression were observed, likely due to a low sample number (3, 31). In other tumors, its expression has been reported low as in renal cell carcinoma (32), lung cancer (33), and hepatocellular carcinoma (23); moreover, overexpression of this miRNA in these tumors inhibits cell proliferation, migration, and invasion, leading to it being characterized as a tumor suppressor.

Finally, miR-10a and miR-181a showed no significant difference among any evaluated groups, both on histology (favorable or unfavorable) type and metastasis development; thereby, these can be discarded as possible markers. However, they can be studied more because of the TLDA results in this study, which indicates expression differences, and information reported previously in other neoplasia. MiR-10a-5p dysregulation has not previously been reported for WTs. However, it has been reported to promote metastasis and EMT (34). In EMT, differentiated cells gradually dedifferentiate and undergo phenotypic changes, thereby promoting tumor malignancy.

Regarding miR-181a, dysregulation of miR-181 family members has been reported for different types of cancer, such as small cell lung cancer with VCAM-1, an important factor in cell migration and invasion, being one of their targets. In this study, DAWT tissue exhibited low miR-181 expression, which would be expected to lead to reduced VCAM-1 inhibition, thereby increasing the likelihood of tumor cell invasion and migration in DAWT (35). These observations are in agreement with estimates indicating that 50% of patients with DAWT develop metastasis (36).

Other clinicopathological characteristics analyzed were the cell types that make up the tumor and which are considered important prognostic factors (37). Blastemal cells were the largest histological classification group identified in the studied cases (55%). However, there was no significant difference in miRNA expression between triphasic and blastemal samples, suggesting that changes in miRNA levels affect all the WT cell types equally, except mesenchymal cells. ISH results showed that mesenchymal cells had the lowest expression of the three evaluated miRNAs. This could be explained by the fact that these cells are intermediates in the transition between the less differentiated blastemal cells and the most differentiated epithelial cells. Furthermore, mesenchymal cells are the scarcest cell type present in WT and are found only rarely in clusters with mature epithelial cells (38). In terms of tumor stage, several studies associate metastasis with the EMT process in more advanced stages (39). Nevertheless, we found only differences between tumor stage III and the control group for miR-29a.

For the comparison among the different age ranges, only miR-29a and miR-218 showed significant differences between the control group and the two younger groups, so this expression difference coincides with ages related to the modification process in kidney development. These age ranges are coherent with the most frequently reported age range for WT, 5 years old and under (1).

In our work, we did not find differences between pretreated and non-pretreated tumors in any miRNAs, so the miRNA expression is independent, and they are not affected by any surgical treatment. It would be interesting to investigate miRNA changes in WT-related secondary tumors; nevertheless, most of them are discarded due to the tumor cell dissemination of high risk, concluding on complicated biopsies (40).

From the MLPA trial, an alteration in the number of copies in various genes was observed, highlighting the *WTX* gene (*AMER1*), which previously in the literature was reported as having mutated from 18 to 29% in WT (41). In contrast, in this study, we observed a higher percentage of cases (47%) with this gene deletion. This difference in *WTX* frequency could be related to ethnicity. This result demonstrated *WTX* participation in WT development (41). *WTX* can be considered to be a master regulator in WT, for its percentage of altered cases and for its different genes and miRNAs with which it interacts.

In this study, TP53 was observed with a diminished number of copies in four cases out of 19 (21%); patients with this alteration have an unfavorable prognostic. This result contrasts with data reported in the literature, which shows five percent of cases (41). Andrade and his collaborators in 2013 associated *TP53* mutation with DAWT due to its poor prognostic; however, only two of the four cases with an alteration in this gene belong to this histological tissue. Many times molecular alterations are not reflected in histological types of tissue in WT (42). Wegert et al. in 2017 demonstrated that not all types of WT with *TP53* mutation are anaplastic tumors, due to the fact that they mostly do not meet the criteria previously mentioned in this work, so it is suggested that *TP53* can be part of the development of anaplastic tissue, but they are not always present in DAWT (43, 44).

**Figure 10:**
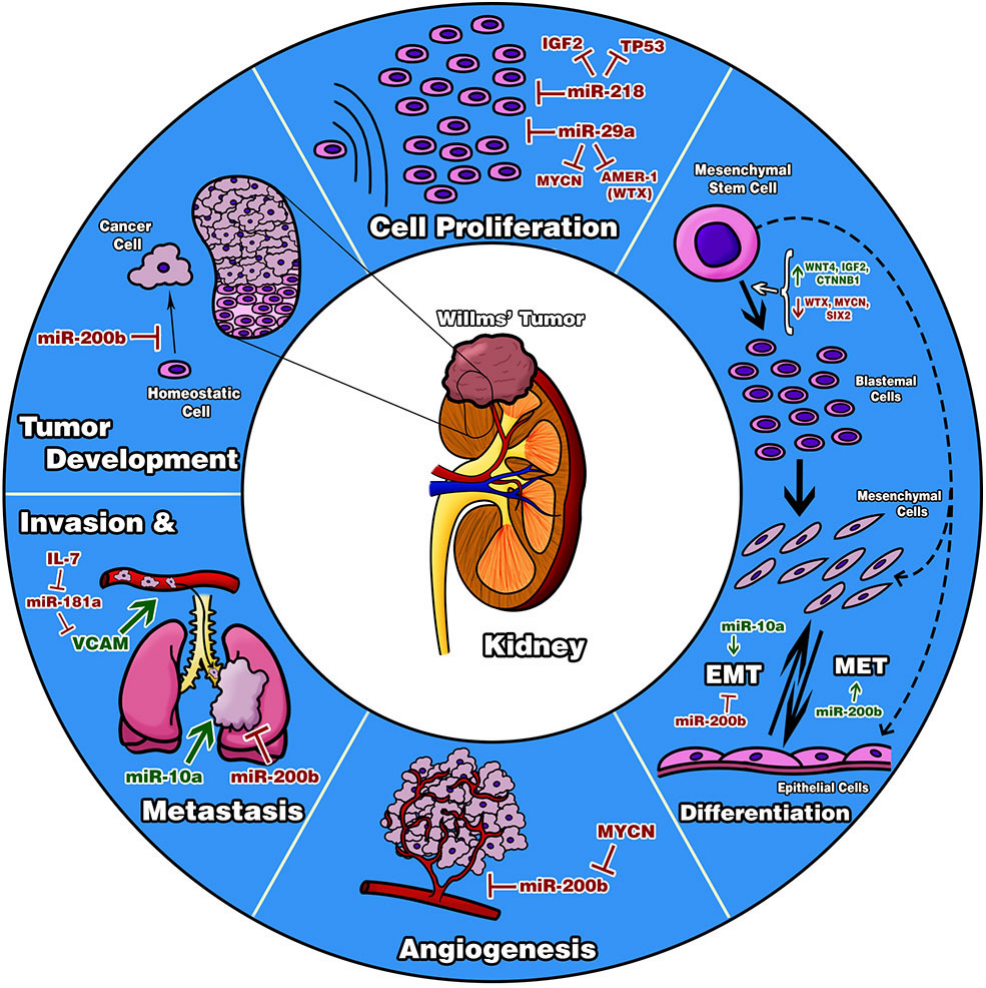
Diagram of biological processes proposed to be involved in Wilms tumor. The diagram describes important processes reported in other neoplasia types in which our evaluated miRNAs are involved; thereby, it is suggested that these five miRNAs are participating the same way in Wilms tumor inhibiting or favoring these processes. It is important to highlight that these probable processes are only speculative. Functional studies with tumor cell lines will be necessary to confirm our results.

The importance of the study of the variation in the number of copies in some genes is implicated in some pathways of WT development or regulates some important miRNAs involved in WT as well. In this way, it was searched for genes that regulate the studied miRNAs, through the “TransmiR” database (17). In the case of miR-10a and miR-200b, they can be activated by TP53, so that WT with a low expression in this gene can show alteration in the expression of both miRNAs. As opposed, *TP53* suppresses miR-29a expression; this miRNA inhibits the MYC gene. In our study, we found that miR-200b is located in the 1p36.33 region which is altered in three cases (16%). Talking about miR-181a and miR-218, it was known that *MYCN* induces its transcription so that the gene can be a factor for its expression alterations. Every miRNA and gene studied are involved in a biologic process and in the development of this neoplasia, so it is relevant to study them together (Figure 10).

In accord with the convergence pathway of the five evaluated miRNAs through *in silico* analyses, it can be found that the Hippo signaling pathway is a deep fully described cancer development pathway due to its fundamental role in cell proliferation, tumorigenesis, organ development, and apoptosis regulation (45). One main effector protein on the Hippo signaling pathway is the transcriptional coactivator YAP1 (yes-associated protein-1). In WT, YAP1 is upregulated mainly in DAWT tissues (46). In the same way, YAP1 inhibition by miRNAs has been studied in other tumors, such as gastric, breast, and thyroid (47). Also, YAP1 can interact with ZEB1 in the EMT process, which favors tumor progression and metastasis in WT (48).

In the Hippo signaling pathway, there are about 19 central genes with oncogenic and tumor suppressor activities that can interact with studied miRNAs (49); in this way, the miR-200 family was highlighted as a YAP1 master regulator (50). It is important to consider that simultaneously miR-200b also regulates ZEB1, and together they can take part in WT development. In the WT context, there is not much information about the contribution of this signaling pathway, but it can be a great target for future studies.

In conclusion, WT is a heterogeneous cancer (20), in which epigenetic alterations influence its biologic behavior. In this study, several miRNAs were found to be differentially expressed between non-neoplastic kidney tissue and WT tissue, and these differences were especially pronounced in DAWT and metastatic tumor tissues. The relationship between mutated genes and miRNA expression changes in this pathology is complex; however, it allows for the identification of potential biomarkers among the affected molecular pathways. In our case, we found that five identified and analyzed miRNAs are common participants in the Hippo pathway, while four are involved in nine other cancer-related processes, including PI3k/Akt signaling, adherent junctions, and the TP53 signaling pathway.

## Data Availability Statement

The datasets generated for this study are available on request to the corresponding author.

## Ethics Statement

The studies involving human participants were reviewed and approved by Comité de Ética del Hospital Infantil de México Federico Gómez. Written informed consent from the participants' legal guardian/next of kin was not required to participate in this study in accordance with the national legislation and the institutional requirements.

## Author Contributions

PE-A and MP-D established the protocol. FP-L, JG-Q, and PE-A designed the experiments. CS-B examined the ISH data. LC-M and SS-P examined the WT slices and corroborated the anaplastic diagnosis. PE-A and FP-L analyzed the results. GB-G did the scanning and quantification. MM-M was responsible for the clinic pathologic database contribution. AC-R performed the *in silico* analyses. FP-L and PE-A wrote the manuscript.

## Conflict of Interest

The authors declare that the research was conducted in the absence of any commercial or financial relationships that could be construed as a potential conflict of interest.

## Supplementary Material

Supplementary Figure 1 220 miRNAs global expression was observed on four anaplastic WT and eight non-anaplastic WT by TLDA. Red indicates increased expression, black indicates no change in expression, and green indicates reduced expression. The map was generated using MeV software.

Supplementary Figure 2 MiRNAs expression comparison between DAWT, FHWT, controls, metastatic, and non-metastatic groups through RT-qPCR. In these graphics a comparison between DAWT, and FHWT were shown for miR-10a **(B)**, miR-181a **(D)**, and miR-200b **(E)**, where it was observed no significant differences on the expression in any evaluated groups. Also it was observed the miR-10a **(A)**, and miR-181a **(C)** comparisons between metastatic, and non-metastatic groups, no significant differences were found. Kruskal-Wallis tests with Dunn's *post-hoc* were performed using *P* < 0.05.

Supplementary Figure 3 MiRNAs expression values comparison between preoperative chemotherapy, and without preoperative chemotherapy patients through RT-qPCR. There are no significant differences between chemotherapy conditions in miR-10a **(A)**, miR-181a **(C)** and miR-200b **(D)**. However, miR-29a **(B)** showed differences between the control group and the group without preoperative chemotherapy with *p* = 0.0074, whereas miR-218 **(E)** showed differences between the control group and the chemotherapy conditions. Kruskal-Wallis tests with Dunn's *post-hoc* were performed using *P* < 0.05.

Supplementary Figure 4 MiRNAs relative expression comparison between male and female patients through RT-qPCR. It was observed miR-181a **(C)**, and miR-218 **(E)** significant differences between controls, and male gender with *p* = 0.0255, and *p* = 0.0005 respectively, whereas miR-29a **(B)** showed significant differences between the control group and female gender with *p* = 0.0016. MiR-10a **(A)**, and miR-200b **(D)** showed no significant differences between any groups. Kruskal-Wallis tests with Dunn's *post-hoc* were performed using *P* < 0.05.

Supplementary Figure 5 MiRNAs relative expression comparison among the five tumor stages in WT through RT-qPCR. For miR-10a **(A)**, miR-181a **(C)**, miR-200b **(D)**, and miR-218 **(E)** there were no significant differences between any groups. Only miR-29a **(B)** showed significant differences between the control group and stage III with *p* = 0.03355. Kruskal-Wallis tests with Dunn's *post-hoc* were performed using *P* < 0.05.

Supplementary Figure 6 MiRNAs expression comparison between WT patients' age ranges through RT-qPCR. There are no significant differences among age ranges in miR-10a **(A)**, miR-181a **(C)**, and miR-200b **(D)**, whereas miR-29a **(B)**, and miR-218 **(E)** showed significant difference with one age range and the control group. Kruskal-Wallis tests with Dunn's *post-hoc* were performed using *P* < 0.05.

Supplementary Figure 7 Expression values comparison between the two more frequent WT histology types through RT-qPCR. For miR-10a **(A)**, miR-181a **(C)**, and miR-200b **(D)** there were no significant differences between evaluated groups, whereas miR-29a **(B)** showed significant differences compared to control, and miR-218 **(E)** only showed differences between blastemal and the control groups. Kruskal-Wallis tests with Dunn's *post-hoc* were performed using *P* < 0.05.

Supplementary Figure 8 Biological pathways in which 109 expressed miRNAs are involved (667), based on the mirPath v.3 database (DIANA TOOLS). Red color indicates the pathways where miRNAs have low (668) significant values, beige color indicates no differences. In the dendrogram, the x-axis label indicates the (669) hierarchical grouping of the miRNAs. The seven main pathways identified were the p53 signaling (667) pathway, viral carcinogenesis, cell cycle, hepatitis B infection, prostate cancer, cancer pathways, and bladder (671) cancer.

Supplementary Figure 9 Metastatic and non-metastatic intensity values comparison through ISH assay. It can be observed both for PT analysis (top row), and N analysis (bottom row) there are only significant differences between non-metastatic cases and their respectives control groups with values of *p* = 0.0020, and *p* = 0.0001 for miR-181a; *p* = 0.0023, and *p* = 0.0006 for miR-200b; and *p* = 0.0004, and *p* < 0.0001 for miR-218. Kruskal-Wallis tests with Dunn's *post-hoc* were performed using *P* < 0.05.

Supplementary Figure 10 Comparison between the three cell types inside the triphasic phenotype and its miRNAs expression in WT through ISH. There were no significant differences between any WT cell type on evaluated miRNAs: miR-181a **(A)**, miR-200 **(B)**, and miR-218 **(C)**. Kruskal-Wallis tests with Dunn's *post-hoc* were performed using *P* < 0.05.
