# Supplementary figures and images for: MicroRNA Profiling in Wilms Tumor: Identification of Potential Biomarkers
Source: Front Pediatr. 2020 Jul 16;8:337. doi: 10.3389/fped.2020.00337 (PMC7378594; doi:10.3389/fped.2020.00337)

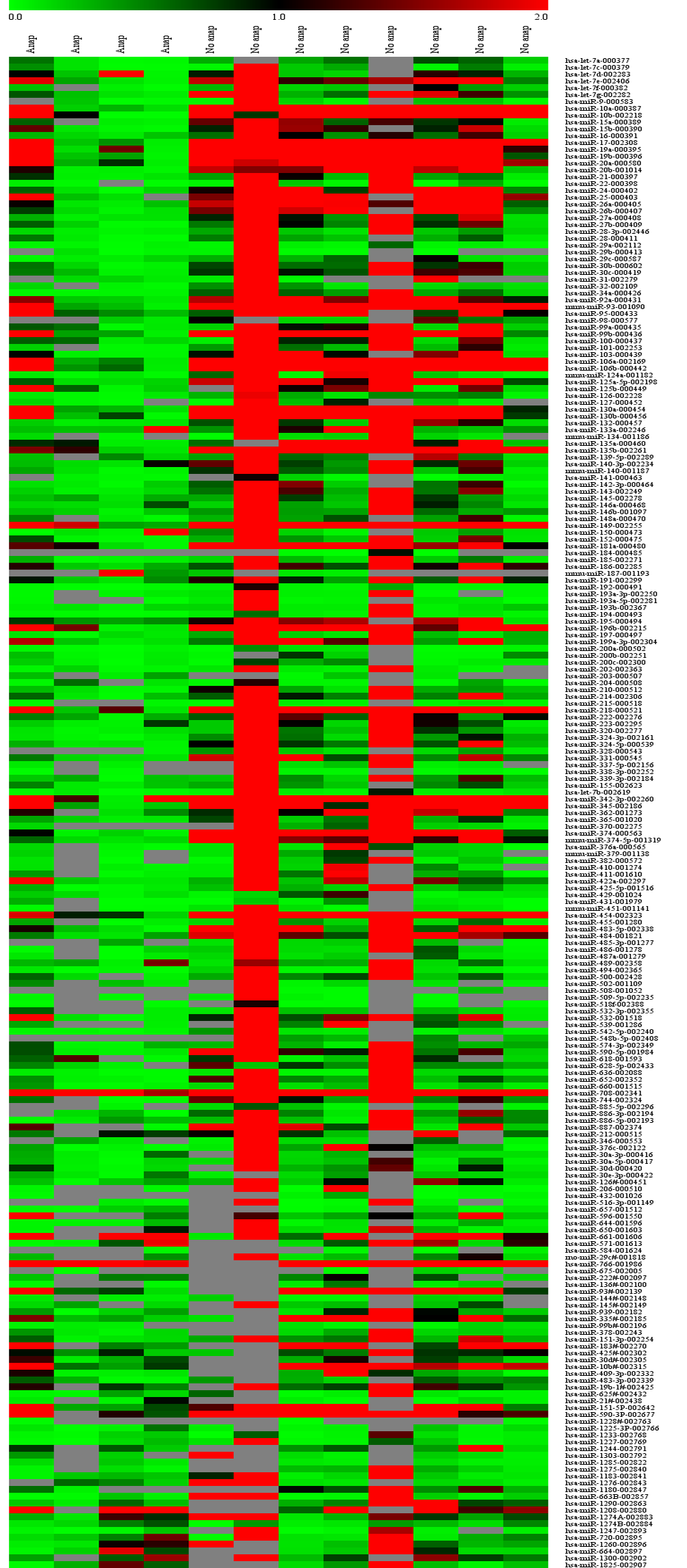

Supplement: Supplementary Figure 1 — 220 miRNAs global expression was observed on four anaplastic WT and eight non-anaplastic WT by TLDA. Red indicates increased expression, black indicates no change in expression, and green indicates reduced expression. The map was generated using MeV software. [file Image_1.TIFF]

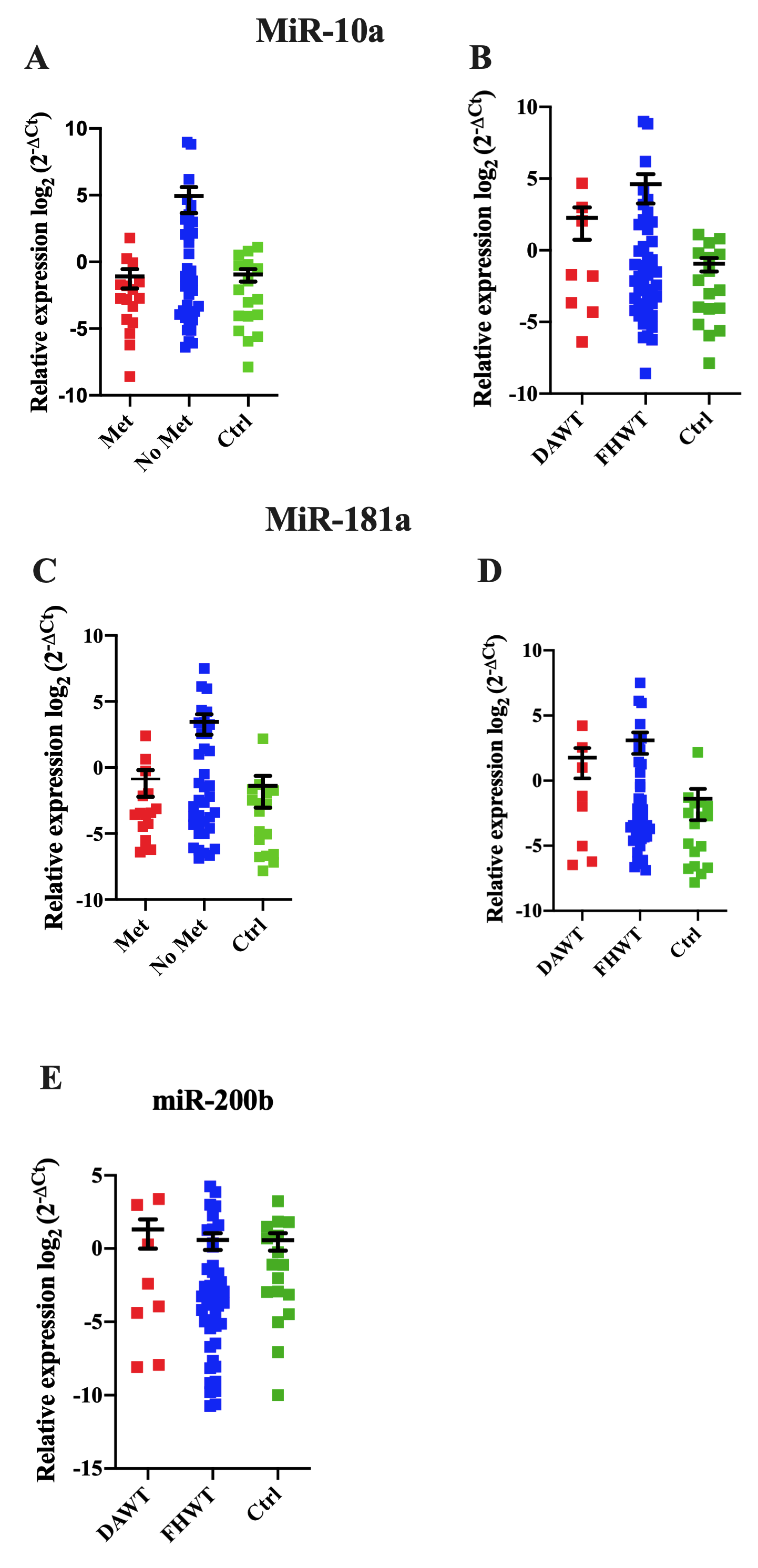

Supplement: Supplementary Figure 2 — MiRNAs expression comparison between DAWT, FHWT, controls, metastatic, and non-metastatic groups through RT-qPCR. In these graphics a comparison between DAWT, and FHWT were shown for miR-10a (B), miR-181a (D), and miR-200b (E), where it was observed no significant differences on the expression in any evaluated groups. Also it was observed the miR-10a (A), and miR-181a (C) comparisons between metastatic, and non-metastatic groups, no significant differences were found. Kruskal-Wallis tests with Dunn's post-hoc were performed using P < 0.05. [file Image_2.TIFF]

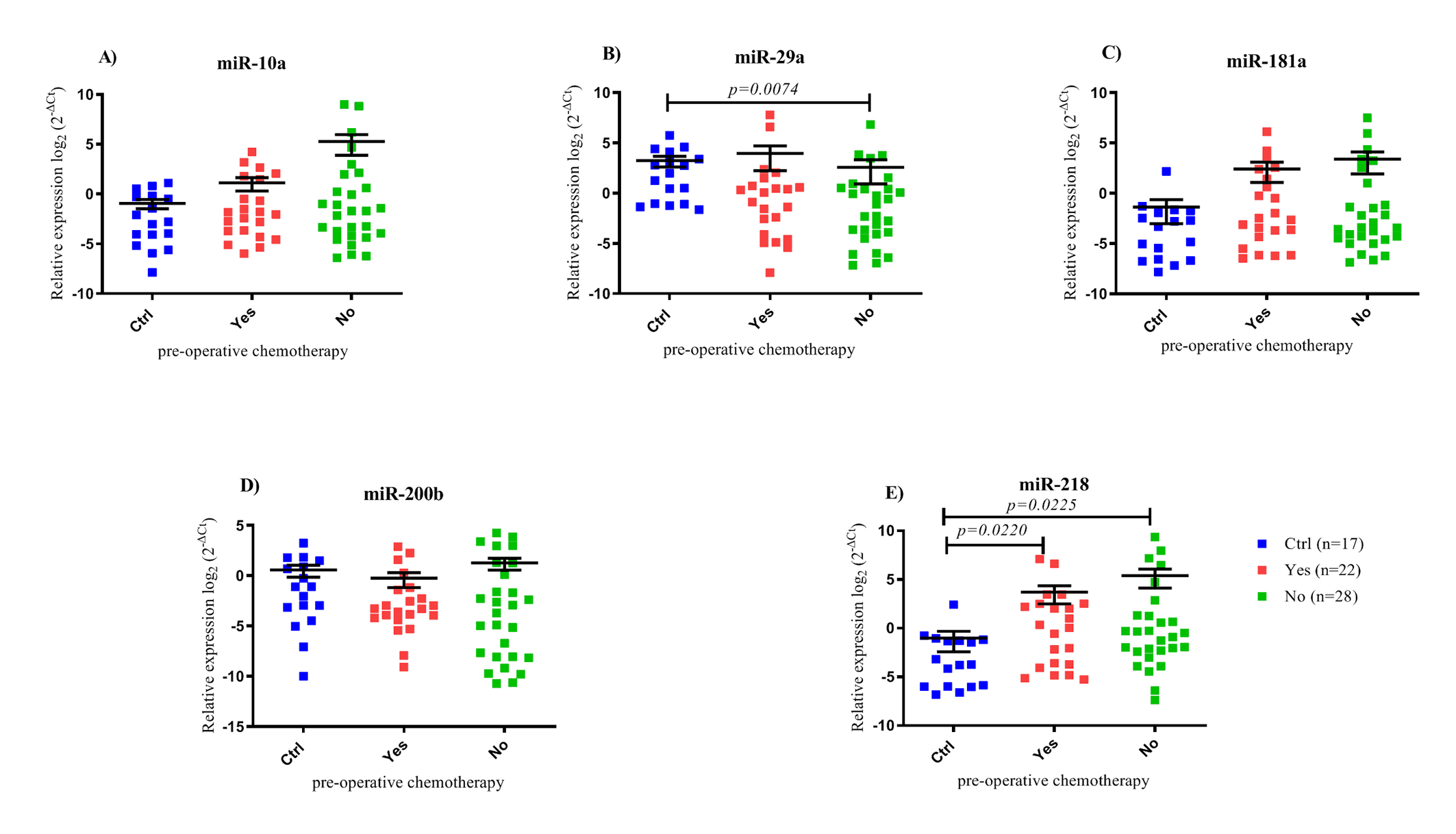

Supplement: Supplementary Figure 3 — MiRNAs expression values comparison between preoperative chemotherapy, and without preoperative chemotherapy patients through RT-qPCR. There are no significant differences between chemotherapy conditions in miR-10a (A), miR-181a (C) and miR-200b (D). However, miR-29a (B) showed differences between the control group and the group without preoperative chemotherapy with p = 0.0074, whereas miR-218 (E) showed differences between the control group and the chemotherapy conditions. Kruskal-Wallis tests with Dunn's post-hoc were performed using P < 0.05. [file Image_3.TIF]

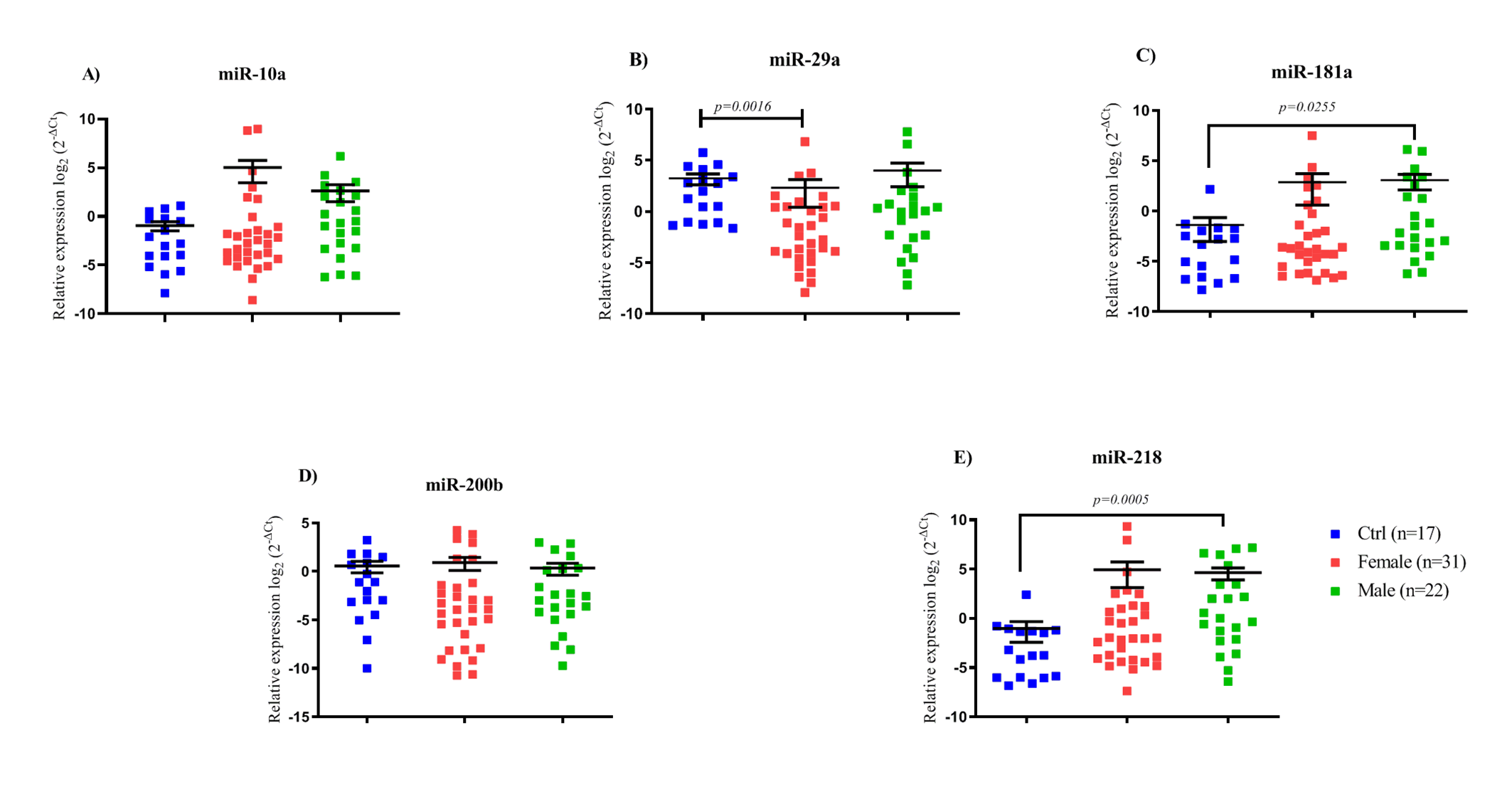

Supplement: Supplementary Figure 4 — MiRNAs relative expression comparison between male and female patients through RT-qPCR. It was observed miR-181a (C), and miR-218 (E) significant differences between controls, and male gender with p = 0.0255, and p = 0.0005 respectively, whereas miR-29a (B) showed significant differences between the control group and female gender with p = 0.0016. MiR-10a (A), and miR-200b (D) showed no significant differences between any groups. Kruskal-Wallis tests with Dunn's post-hoc were performed using P < 0.05. [file Image_4.TIF]

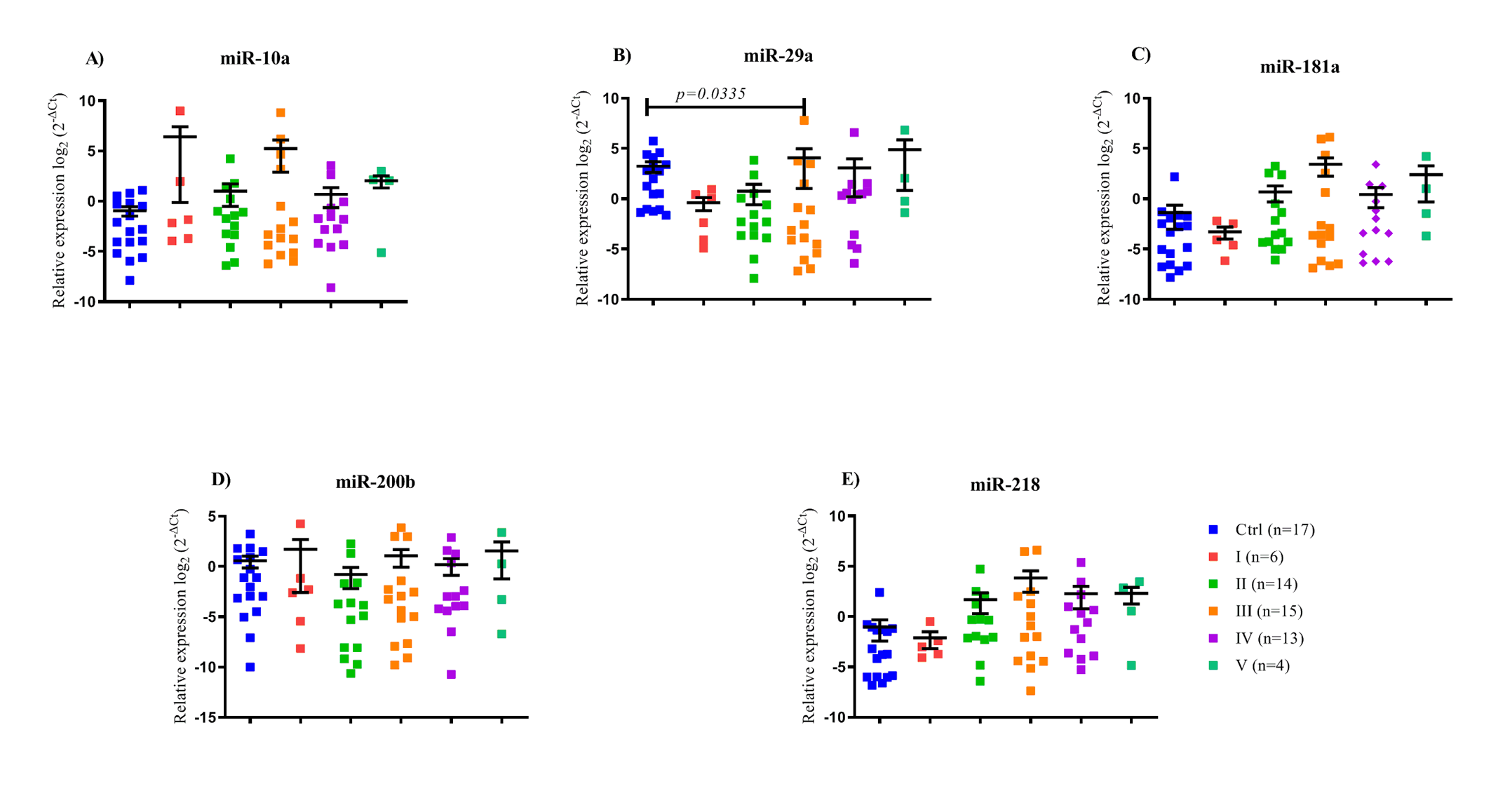

Supplement: Supplementary Figure 5 — MiRNAs relative expression comparison among the five tumor stages in WT through RT-qPCR. For miR-10a (A), miR-181a (C), miR-200b (D), and miR-218 (E) there were no significant differences between any groups. Only miR-29a (B) showed significant differences between the control group and stage III with p = 0.03355. Kruskal-Wallis tests with Dunn's post-hoc were performed using P < 0.05. [file Image_5.TIF]

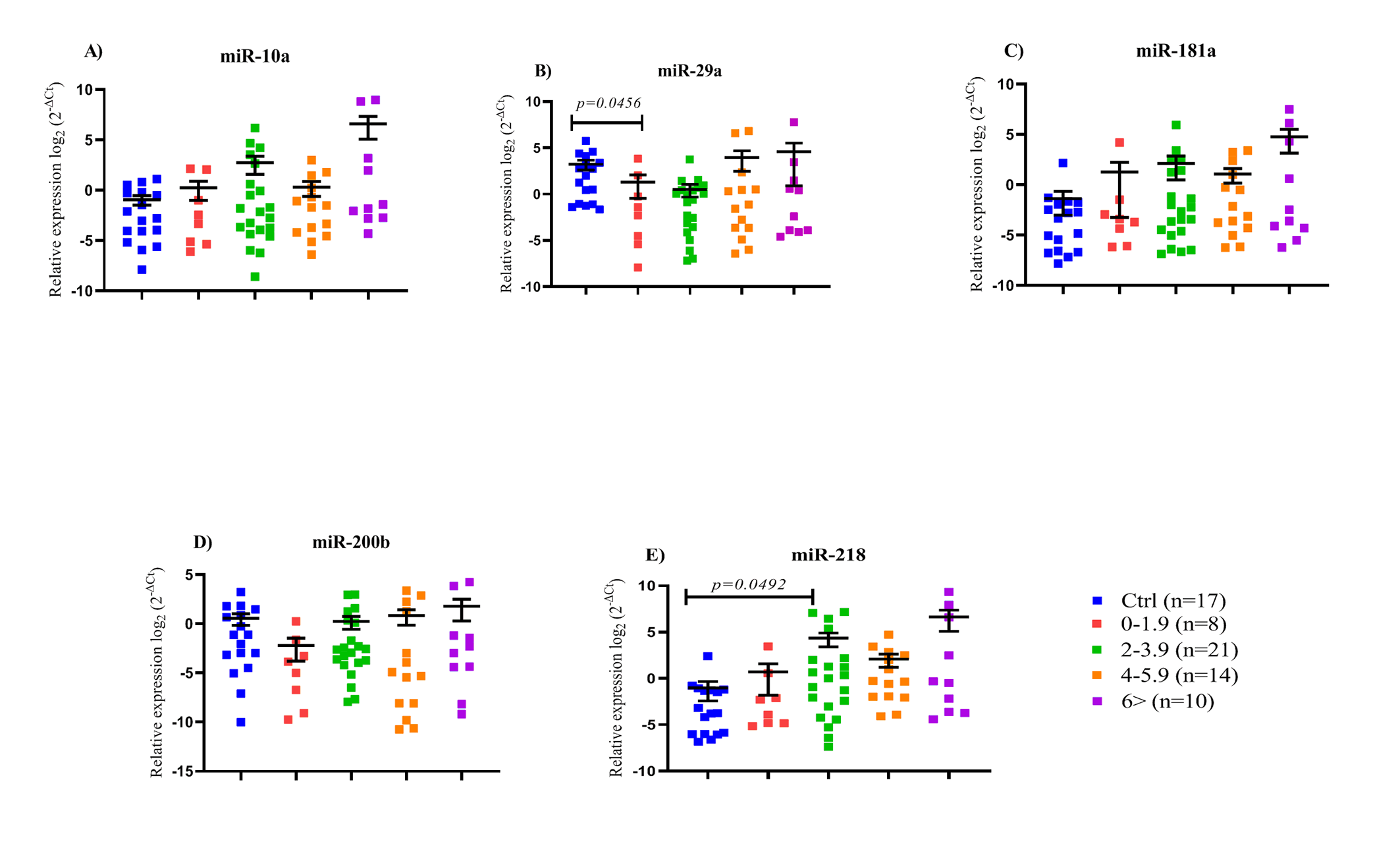

Supplement: Supplementary Figure 6 — MiRNAs expression comparison between WT patients' age ranges through RT-qPCR. There are no significant differences among age ranges in miR-10a (A), miR-181a (C), and miR-200b (D), whereas miR-29a (B), and miR-218 (E) showed significant difference with one age range and the control group. Kruskal-Wallis tests with Dunn's post-hoc were performed using P < 0.05. [file Image_6.TIF]

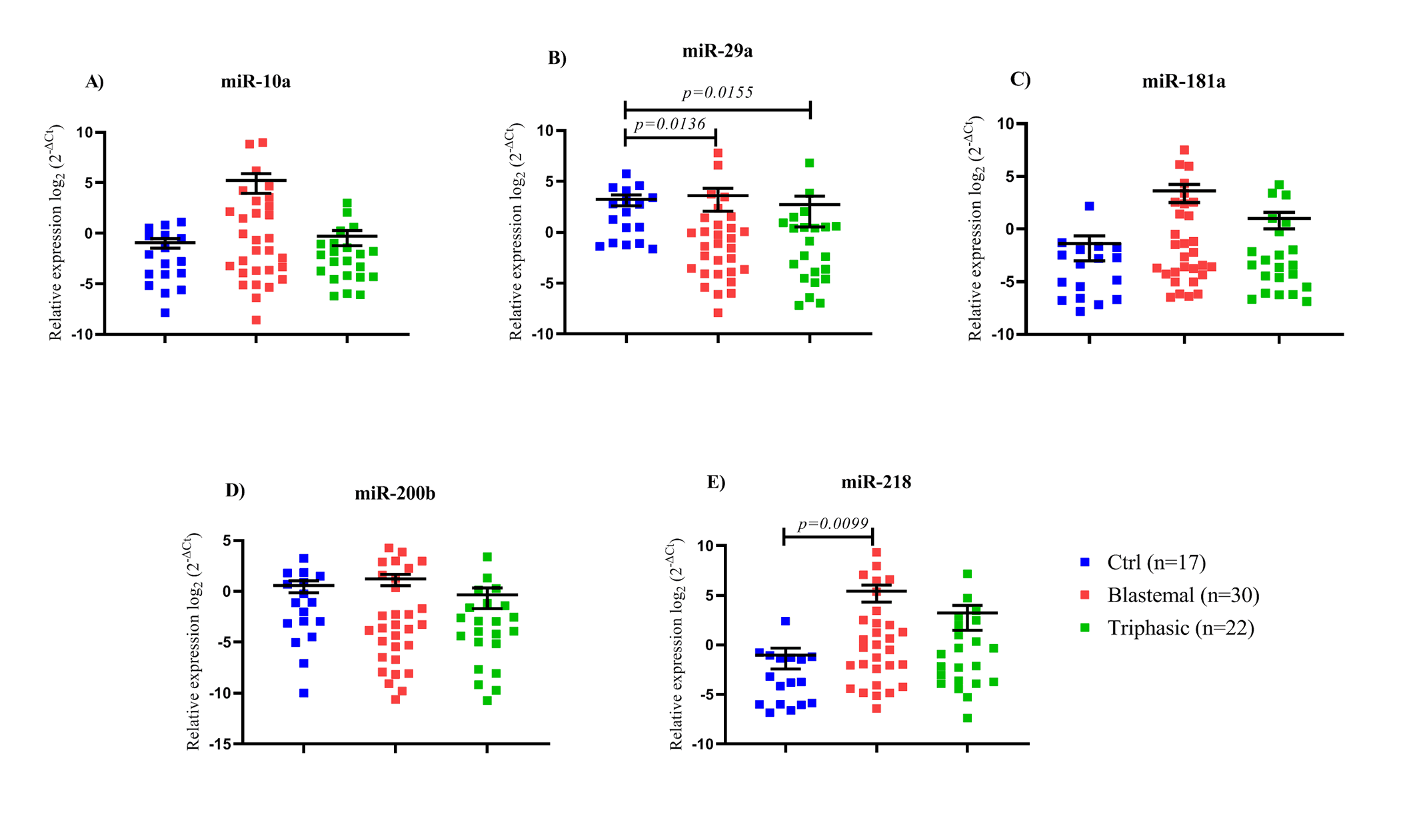

Supplement: Supplementary Figure 7 — Expression values comparison between the two more frequent WT histology types through RT-qPCR. For miR-10a (A), miR-181a (C), and miR-200b (D) there were no significant differences between evaluated groups, whereas miR-29a (B) showed significant differences compared to control, and miR-218 (E) only showed differences between blastemal and the control groups. Kruskal-Wallis tests with Dunn's post-hoc were performed using P < 0.05. [file Image_7.TIF]

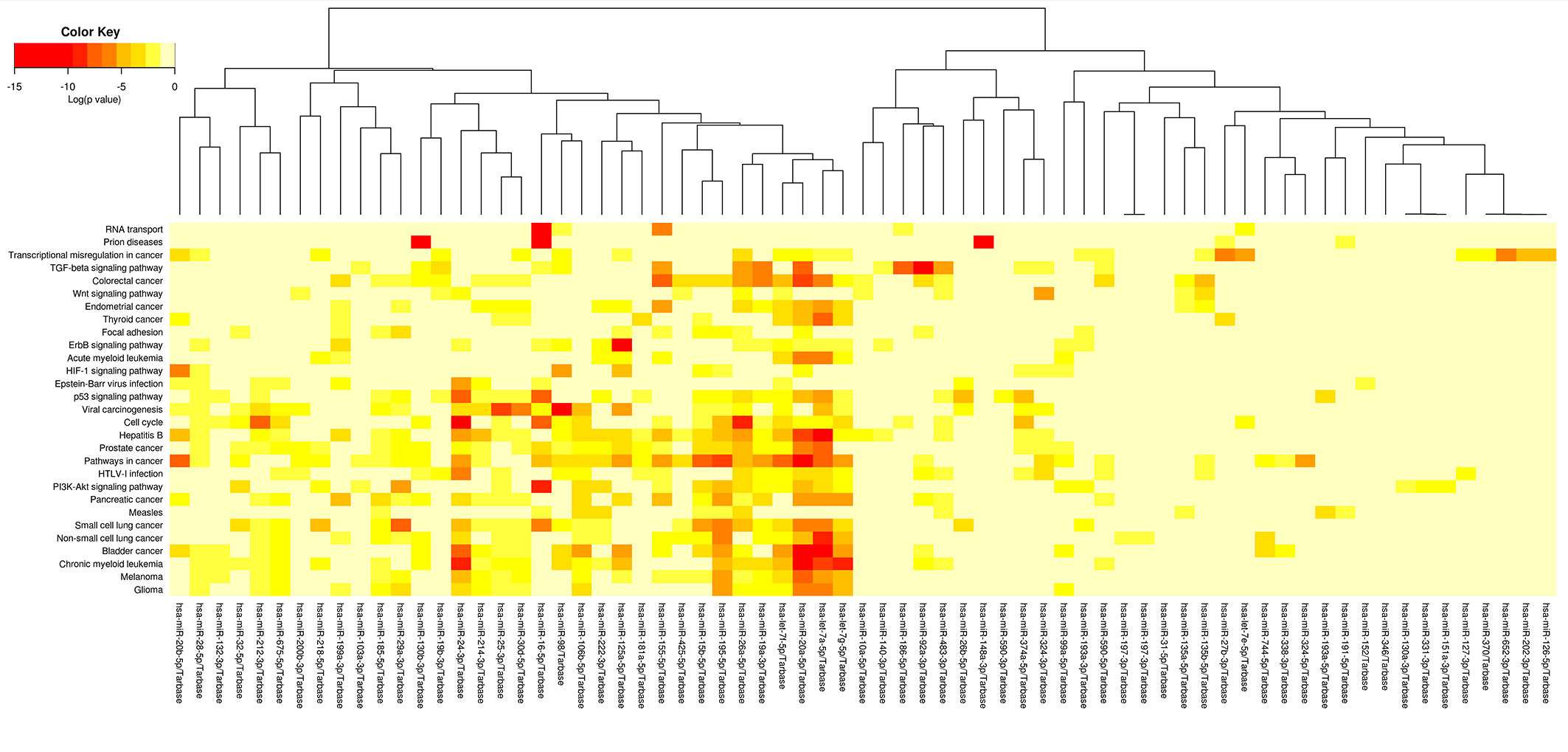

Supplement: Supplementary Figure 8 — Biological pathways in which 109 expressed miRNAs are involved (667), based on the mirPath v.3 database (DIANA TOOLS). Red color indicates the pathways where miRNAs have low (668) significant values, beige color indicates no differences. In the dendrogram, the x-axis label indicates the (669) hierarchical grouping of the miRNAs. The seven main pathways identified were the p53 signaling (667) pathway, viral carcinogenesis, cell cycle, hepatitis B infection, prostate cancer, cancer pathways, and bladder (671) cancer. [file Image_8.TIF]

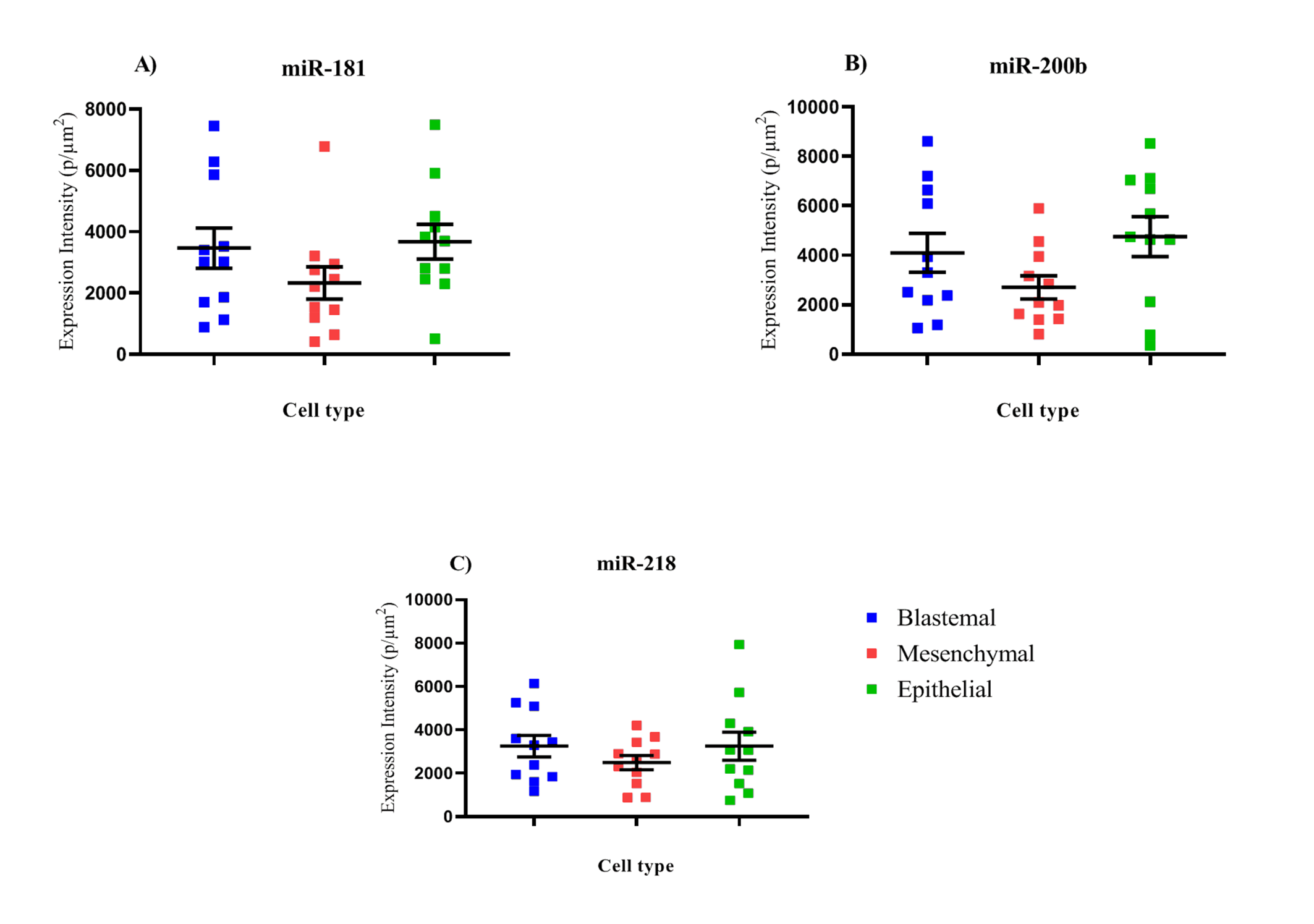

Supplement: Supplementary Figure 10 — Comparison between the three cell types inside the triphasic phenotype and its miRNAs expression in WT through ISH. There were no significant differences between any WT cell type on evaluated miRNAs: miR-181a (A), miR-200 (B), and miR-218 (C). Kruskal-Wallis tests with Dunn's post-hoc were performed using P < 0.05. [file Image_10.TIF]
